# Heteroatom-Engineered Covalent Organic Frameworks Break the CO_2_ Separation Trade-Off in Mixed Matrix Membranes

**DOI:** 10.1021/jacs.5c23169

**Published:** 2026-05-21

**Authors:** Tsukasa Irie, Liting Yu, Sourav Ghosh, Mika Nozaki, Kohki Sasaki, Tokuhisa Kawawaki, Ranjit Thapa, Yu Zhao, Saikat Das, Zixi Kang, Yuichi Negishi

**Affiliations:** † Institute of Multidisciplinary Research for Advanced Materials, 13101 Tohoku University, 2-1-1 Katahira, Aoba-ku, Sendai 980-8577, Japan; ‡ Shandong Key Laboratory of Intelligent Energy Materials, School of Materials Science and Engineering, China University of Petroleum (East China), Qingdao, Shandong 266580, PR China; § Department of Physics, SRM University−AP, Amaravati 522 240, Andhra Pradesh, India; ∥ Centre for Computational and Integrative Sciences, SRM University−AP, Amaravati 522 240, Andhra Pradesh, India; ⊥ Zhejiang Engineering Laboratory for Green Syntheses and Applications of Fluorine-Containing Specialty Chemicals, Institute of Advanced Fluorine-Containing Materials, 66344 Zhejiang Normal University, 321004 Jinhua, China; # State Key Laboratory of Heavy Oil Processing, China University of Petroleum (East China), Qingdao, Shandong 266580, PR China

## Abstract

Breaking the long-standing permeability–selectivity trade-off remains a central challenge in membrane-based carbon dioxide separations. Here we report a heteroatom-engineering strategy that leverages structurally precise covalent organic frameworks (COFs) to transcend this limitation in mixed matrix membranes (MMMs). Two isostructural, π-conjugated two-dimensional COFs, TUS-621 and TUS-622, were rationally designed through symmetry-guided reticulation of a hexatopic triphenylene node with oxygen- and sulfur-containing diamine linkers, respectively, enabling systematic modulation of pore surface chemistry without altering topology. When incorporated into a Pebax polymer matrix, these COFs function as CO_2_-philic, molecularly defined transport domains that synergistically couple preferential CO_2_ sorption with ordered and fast diffusion channels. The optimized TUS-621/Pebax-10% membrane exhibits a CO_2_ permeability of 433 Barrer with a CO_2_/CH_4_ selectivity of 55.3 under mixed-gas conditions, decisively surpassing the 2008 Robeson upper bound for CO_2_/CH_4_ separation while simultaneously achieving high CO_2_/H_2_ separation performance (CO_2_ permeability of 407 Barrer and selectivity of 25.2). Comprehensive pressure- and temperature-dependent permeation studies reveal that selectivity remains remarkably stable over 2–10 bar and 25–100 °C, underscoring the robustness of the COF-enabled transport pathways. Long-term operation over 30 days shows negligible performance decay, highlighting excellent resistance to physical aging and interfacial degradation. Comparative analysis establishes that oxygen-rich pore environments in TUS-621 impart stronger CO_2_ affinity and higher accessible surface area than the sulfur-containing analogue, directly translating molecular-level design into macroscopic separation performance. This work demonstrates that heteroatom-engineered COFs provide a powerful platform for overcoming fundamental transport trade-offs and advancing MMMs toward practical, high-efficiency CO_2_ separations.

## Graphical abstract

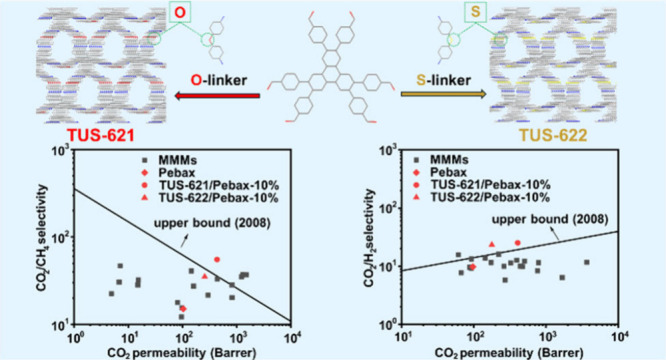

## 1 Introduction

The efficient separation of carbon dioxide from gas mixtures such as CO_2_/CH_4_ and CO_2_/H_2_ lies at the heart of multiple energy- and climate-critical technologies, spanning natural gas upgrading, hydrogen purification, syngas processing, and emerging carbon management strategies.^1–5^ In natural gas streams, CO_2_ must be removed to enhance calorific value, prevent pipeline corrosion, and meet transportation standards, while in hydrogen production and purification, CO_2_ separation is essential to enable high-purity H_2_ for ammonia synthesis, fuel cells, and future hydrogen economies.^6–9^ Conventional separation technologies—amine scrubbing,^10^ cryogenic distillation,^11^ and pressure swing adsorption^12^—remain energy-intensive, capital-heavy, and operationally complex, motivating the development of membrane-based alternatives^13^ that promise lower energy consumption, modularity, and continuous operation. Membrane separations, however, are fundamentally constrained by the long-recognized permeability–selectivity trade-off, empirically captured by the Robeson upper bound,^14,15^ which defines a performance envelope for polymeric membranes beyond which simultaneous gains in permeability and selectivity are rarely achieved. This limitation is particularly acute for CO_2_/CH_4_ and CO_2_/H_2_ separations, where large differences in molecular size, polarizability, and quadrupole moment must be exploited without sacrificing throughput. For practical deployment, membranes must therefore deliver not only high selectivity but also sufficiently high permeability to minimize membrane area and capital cost—requirements that are rarely met simultaneously.

In response, extensive efforts have been devoted to the development of CO_2_-philic materials, including metal–organic frameworks (MOFs),^16^ covalent organic frameworks (COFs),^17^ porous organic polymers,^18^ ionic liquids,^19^ graphene-based materials,^20^ and hybrid composites.^21^ MOFs, for example, offer high surface areas and tunable chemistry but often suffer from moisture sensitivity and framework instability. Ionic liquids and facilitated transport membranes can enhance CO_2_ solubility but are frequently limited by viscosity, slow kinetics, and long-term stability. Two-dimensional (2D) materials such as graphene oxide introduce fast diffusion pathways but typically rely on defect-mediated transport that compromises selectivity control. Within this landscape, reticular materials^22^—particularly COFs^23–38^ —have emerged as uniquely attractive candidates for gas separation due to their crystalline order, permanent porosity, modular chemical design, and exceptional structural robustness. Unlike amorphous porous polymers, COFs provide atomically defined pore environments that can be tailored with precision, enabling rational control over gas–framework interactions. Recent studies have demonstrated the promise of COFs for CO_2_ separation;^39–43^ however, translating their intrinsic adsorption selectivity into membrane-level performance remains nontrivial, especially under mixed-gas conditions.

Mixed matrix membranes (MMMs), which combine porous fillers with polymer matrices, represent a compelling strategy to bridge this gap.^44–46^ By embedding selective fillers into processable polymers, MMMs aim to synergistically integrate high permeability, mechanical integrity, and chemical selectivity. Yet, despite notable advances, most reported MMMs for CO_2_/CH_4_ separation still fall short of breaking the Robeson upper bound in a practically meaningful way.^47–52^ For instance, ACOF-1/Matrimid MMMs exhibit a respectable CO_2_/CH_4_ selectivity of 32.4 but a low CO_2_ permeability of only 15.3 Barrer, limiting throughput and scalability.^47^ Conversely, MOF/COF-16/Pebax MMMs achieve very high CO_2_ permeability (815.9 Barrer) but are marred by modest selectivity (20.3), underscoring the persistent difficulty of simultaneously optimizing both metrics.^48^ Similarly, MOF@COF/Psf MMMs^49^ and NUS-2 COF/Ultem MMMs^50^ deliver encouraging selectivities (up to 46.7) but suffer from extremely low CO_2_ permeabilities (≤15 Barrer), rendering them impractical for industrial deployment. The challenge is even more pronounced for CO_2_/H_2_ separation, where the small kinetic diameter and weak polarizability of H_2_ severely limit selectivity.^53–60^ Graphene oxide-based MMMs incorporating ionic liquids^53^ or cross-linked PEO matrices^54^ show high CO_2_ permeabilities (271–474 Barrer) but only modest CO_2_/H_2_ selectivities (5.8–9.9). MOF-based MMMs such as MIL-53/poly­(ionic liquid) systems improve selectivity (13.3) but at the expense of permeability (92.7 Barrer).^55^ Collectively, these studies highlight a clear lacuna: robust MMMs capable of delivering both high permeability and high selectivity for CO_2_/CH_4_ and CO_2_/H_2_ separations under realistic operating conditions remain elusive.

A critical, yet underexplored, dimension in this context is heteroatom engineering of pore environments.^61,62^ While pore size and topology have traditionally dominated COF and MOF design strategies, increasing evidence suggests that the chemical identity of pore-lining heteroatoms plays a decisive role in governing quadrupole–dipole interactions, adsorption enthalpy, and solubility-selective transport. However, isolating heteroatom effects is challenging in most systems due to simultaneous changes in topology, pore size, or framework flexibility. In this work, we address this challenge by introducing a heteroatom-engineering strategy based on isostructural, π-conjugated 2D COFs with invariant topology and systematically varied pore chemistry. Two COFs, denoted TUS-621 and TUS-622, are constructed via symmetry-guided Schiff-base condensation of the hexatopic *D*_3h_-symmetric node 2,3,6,7,10,11-hexakis­(4-formylphenyl)­triphenylene (HFPTP) with the oxygen-containing diamine 4,4′-diaminodiphenyl ether (ODA) and the sulfur-containing diamine bis­(4-aminophenyl) sulfide (ASD), respectively. This design enables direct comparison of oxygen- versus sulfur-rich pore environments without perturbing framework topology or dimensionality. When incorporated into a Pebax matrix, these COFs function as molecularly defined, CO_2_-philic transport domains that couple preferential adsorption with size-selective diffusion. As demonstrated herein, this heteroatom-engineered approach enables MMMs that not only surpass the Robeson upper bound for CO_2_/CH_4_ separation but also deliver outstanding CO_2_/H_2_ separation performance, operational stability, and long-term durability—establishing a new paradigm for the rational design of next-generation CO_2_ separation membranes.

## 2 Results and Discussion

### Symmetry-Guided Reticulation and Crystallographic Elucidation of π-Conjugated COF Architectures

TUS-621 and TUS-622 were rationally designed as 2D, π-conjugated COFs featuring symmetry-matched reticulation and heteroatom-engineered pore environments for CO_2_ separation in MMMs. Both frameworks were constructed via Schiff-base condensation between a hexatopic, *D*_3h_-symmetric triphenylene core, HFPTP, and two closely related ditopic para-phenylenediamine linkers, ODA and ASD (Figure 1a). The combination of a six-connected planar node with linear two-connected linkers enforces strict geometric commensurability, directing the formation of periodic hexagonal lattices in which long-range order and pore uniformity are intrinsically encoded by molecular symmetry rather than relying on postsynthetic organization.

**Figure 1:**
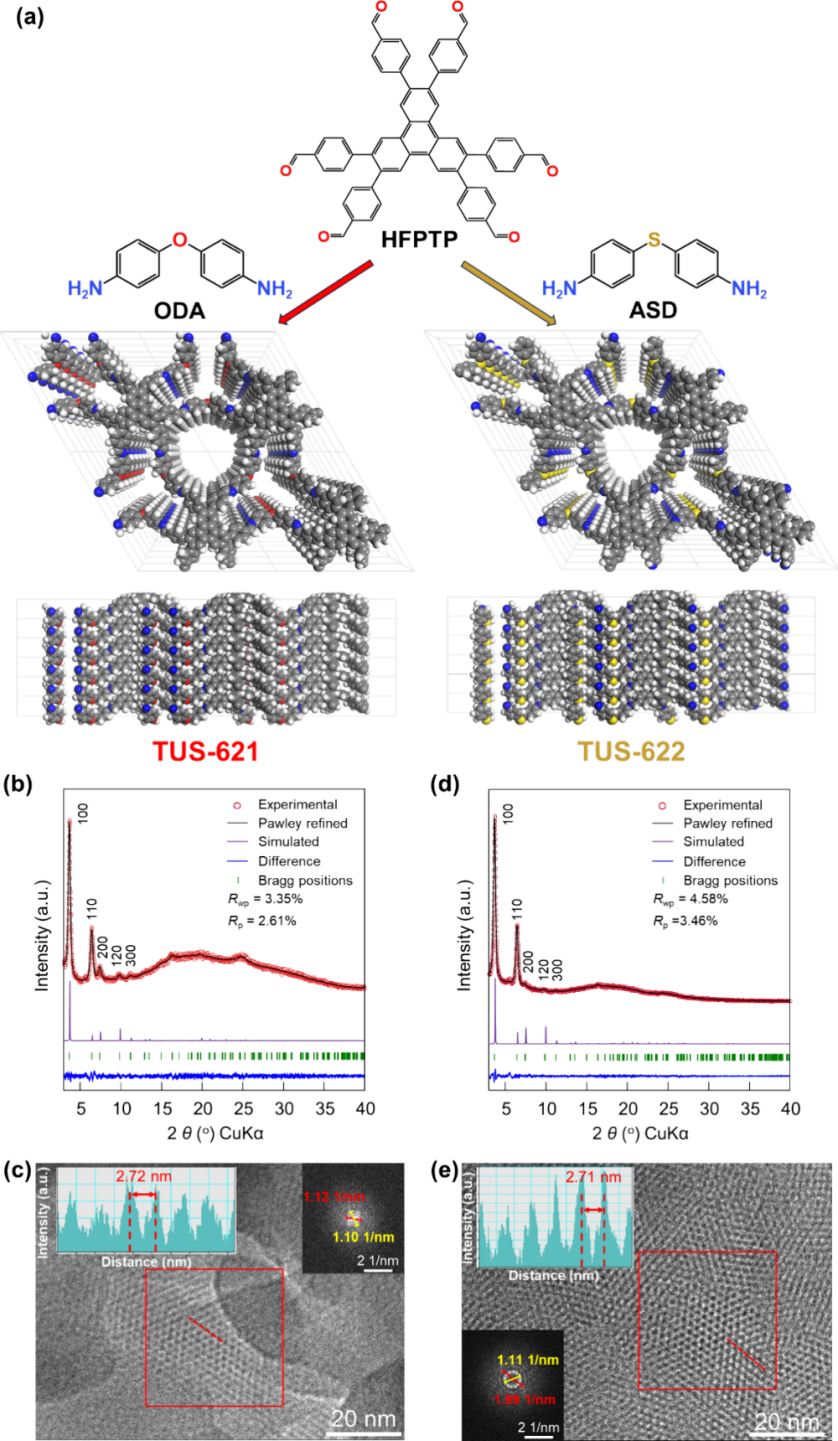
**Structural design and crystallographic characterization of TUS-621 and TUS-622**. (a) Schematic illustration of the symmetry-guided reticulation of the *D*_3h_-symmetric HFPTP node with ditopic ODA and ASD linkers, giving rise to π-conjugated 2D hexagonal COF architectures. (b,d) Experimental (red dots) and simulated (purple traces) PXRD patterns of TUS-621 (b) and TUS-622 (d), showing excellent agreement and confirming the proposed trigonal *P*3 lattice. (c,e) HR-TEM images of TUS-621 (c) and TUS-622 (e), with the corresponding FFT patterns from the marked regions shown in the insets.

The HFPTP unit serves as a rigid π-extended topological vertex that promotes framework planarity, interlayer π–π stacking, and extended conjugation. Beyond structural regularity, the chemical difference between the ODA- and ASD-based linkers is intentionally used to tune CO_2_–framework interactions at the molecular level. In TUS-621, the ether-bridged ODA linker introduces highly electronegative oxygen atoms with localized lone pairs, creating polar microenvironments that enhance CO_2_ sorption through quadrupole–dipole interactions while disfavoring the uptake of less polar CH_4_. In contrast, replacing oxygen with sulfur in TUS-622 increases linker polarizability and soft Lewis basicity, strengthening dispersive and quadrupolar interactions with CO_2_ while subtly modifying diffusion pathways because of the larger van der Waals radius of sulfur. This heteroatom exchange preserves the overall topology and pore geometry while selectively tuning the balance between solubility-driven and diffusion-controlled transport, enabling differential optimization across CO_2_/CH_4_ and CO_2_/H_2_ separations. The rigid π-conjugated backbone defined by HFPTP ensures narrow pore apertures and restricted segmental motion, imparting a pronounced molecular sieving effect that kinetically suppresses larger CH_4_ molecules while maintaining rapid transport of smaller H_2_, whereas the CO_2_-philic pore surfaces selectively amplify CO_2_ permeability through preferential sorption. Collectively, the synergy between symmetry-guided reticulation, heteroatom-engineered pore chemistry, and π-stacked 2D architectures establishes TUS-621 and TUS-622 as structurally precise, chemically tunable platforms ideally suited for high-selectivity CO_2_ separation in MMMs. The crystallographic structures of TUS-621 and TUS-622 were established through a combined experimental–computational workflow integrating powder X-ray diffraction (PXRD) with structural modeling. Force-field geometry optimization performed using the Forcite module (*Materials Studio* 7.0 ^63^) converged to energetically stable 2D frameworks characterized by hexagonal metrics. For TUS-621, the optimized unit cell exhibits parameters of *a* = *b* = 27.1616 Å, *c* = 4.8083 Å, α = β = 90°, and γ = 120°, whereas TUS-622 displays closely related parameters of *a* = *b* = 27.0491 Å, *c* = 4.9290 Å, α = β = 90°, and γ = 120°. Both structures are consistent with a trigonal lattice in the *P*3 space group (No. 143) (Tables S12 and S13). PXRD patterns simulated from the optimized structural models closely reproduce the experimental diffraction profiles, confirming the validity of the proposed frameworks (Figures 1b and 1d). In the experimental PXRD data, TUS-621 shows a series of intense reflections at 3.69°, 6.43°, 7.44°, 9.85°, and 11.18°, while TUS-622 displays analogous peaks at 3.69°, 6.44°, 7.45°, 9.90°, and 11.21°. These reflections are unambiguously indexed to the (100), (110), (200), (120), and (300) crystallographic planes, indicative of well-developed long-range order and periodic pore arrangement within both COFs. Quantitative Pawley refinement further substantiates the structural assignments, yielding excellent agreement factors (*R*_p_ = 2.61%, *R*_wp_ = 3.35% for TUS-621 and *R*_p_ = 3.46%, *R*_wp_ = 4.58% for TUS-622) along with refined lattice parameters (*a* = *b* = 27.1603 Å, *c* = 4.8127 Å for TUS-621; *a* = *b* = 27.0391 Å, *c* = 4.9277 Å for TUS-622) that closely match the optimized models. High-resolution transmission electron microscopy (HR-TEM) images (Figures 1c, 1e, and S9–S10) reveal in-plane periodicities of 2.71 and 2.72 nm, consistent with the *a*/*b* lattice parameters (2.72 nm for TUS-621 and 2.70 nm for TUS-622), indicating the ordered pore arrangement of the frameworks. The corresponding fast Fourier transform (FFT) patterns show diffraction rings at 1.10–1.12 nm^–1^ (*d* ≈ 0.90 nm), which match the (120) reflection derived from PXRD analysis, confirming the crystalline structures of the COFs.

### Morphology, Structure, and Elemental Distribution

The successful construction of imine-linked, π-conjugated frameworks in TUS-621 and TUS-622 was unequivocally confirmed by a combination of vibrational spectroscopy, solid-state NMR, elemental analysis, and gas sorption measurements. Fourier-transform infrared (FT-IR) spectra of both materials display the emergence of intense imine (C=N) stretching bands at 1621 cm^–1^ for TUS-621 and 1622 cm^–1^ for TUS-622, providing clear evidence of Schiff-base condensation. Concomitantly, the characteristic N–H stretching vibrations of the diamine precursors—ODA (3444 and 3386 cm^–1^) and ASD (3408 and 3334 cm^–1^)—as well as the aldehyde C=O stretch of HFPTP at 1699 cm^–1^, are nearly completely extinguished, confirming efficient monomer consumption and high polymerization fidelity (Figures 2a and 2b). Solid-state ^13^C CP/MAS NMR spectra further corroborate covalent framework formation, revealing a distinct resonance centered at approximately 159 ppm for both COFs, which is diagnostic of the imine carbon within an extended conjugated network (Figures 2c and 2d). Elemental analyses are consistent with the proposed framework compositions, yielding measured values of C: 83.72, H: 4.67, N: 5.84, and O: 3.75 for TUS-621 (C_96_H_60_N_6_O_3_) and C: 76.58, H: 5.13, N: 4.59, and S: 7.69 for TUS-622 (C_96_H_60_N_6_S_3_), in close agreement with their respective theoretical values. Minor deviations are attributed to residual adsorbates and the intrinsic limitations of combustion analysis for sulfur-containing frameworks, but overall confirm the expected stoichiometry and high chemical integrity of both materials. Despite sharing identical topology and closely comparable pore dimensions, TUS-621 exhibits a markedly higher Brunauer–Emmett–Teller (BET) surface area than TUS-622. Nitrogen adsorption–desorption collected at 77 K for both TUS-621 and TUS-622 exhibit steep uptake at low relative pressures (*P*/*P*_0_ < 0.1), characteristic of dominant microporosity, accompanied by modest hysteresis loops in the intermediate pressure region, indicative of restricted pore connectivity and framework rigidity (Figure 2e), yielding BET surface areas of 1729 m^2^ g^–1^ for TUS-621 and 846 m^2^ g^–1^ for TUS-622 (Figures S1 and S2). Application of the BETSI protocol affords statistically robust values of 1724 and 767 m^2^ g^–1^, respectively (Figures S5 and S6). This difference likely reflects the distinct chemical nature of the linker heteroatoms rather than major geometric differences. The ether linkage in ODA imparts greater rotational flexibility and reduced steric bulk compared to the sulfide linkage in ASD, facilitating more efficient framework packing, higher accessible internal surface, and reduced pore wall roughness. In contrast, the larger van der Waals radius and higher polarizability of sulfur increase framework mass density and introduce subtle pore surface corrugation, which collectively diminish the fraction of nitrogen-accessible surface area without substantially altering the average pore diameter. Consistent with this interpretation, quenched solid density functional theory (QSDFT) analysis reveals narrowly distributed pore sizes centered at 1.90 nm for TUS-621 and 1.82 nm for TUS-622 (Figures S3 and S4), in excellent agreement with the crystallographically simulated pore apertures. These results confirm that the lower surface area of TUS-622 does not originate from pore collapse or framework disorder, but rather from heteroatom-induced chemical densification. The distinct pore surface chemistries of the two COFs are further reflected in their gas sorption behavior (Figure 2f). At 1 bar, TUS-621 exhibits a CO_2_ uptake of 19.0 cm^3^ g^–1^, significantly exceeding its CH_4_ (3.5 cm^3^ g^–1^) and H_2_ (0.6 cm^3^ g^–1^) uptakes, while TUS-622 adsorbs 14.4 cm^3^ g^–1^ of CO_2_ compared to 5.3 cm^3^ g^–1^ of CH_4_ and 0.2 cm^3^ g^–1^ of H_2_. To further probe framework–gas interactions, the isosteric heats of adsorption (*Q*_st_) were calculated by the virial method (Figure S15 and Table S1). At near-zero coverage, TUS-621 shows *Q*_ST_ values of 18.23, 7.22, and 2.54 kJ mol^–1^ for CO_2_, CH_4_, and H_2_, respectively, while TUS-622 gives 15.18, 9.10, and 1.63 kJ mol^–1^. These results indicate that both frameworks preferentially interact with CO_2_, but that TUS-621 binds CO_2_ more strongly whereas TUS-622 binds CH_4_ more strongly. This trend is consistent with Figure 2f and suggests that the higher CH_4_ affinity of TUS-622 can increase competitive adsorption against CO_2_ in the CO_2_/CH_4_ mixture, thereby contributing to the lower CO_2_/CH_4_ selectivity of TUS-622-based MMMs. In contrast, because H_2_ adsorption is negligible, competitive adsorption effects are minimal in the CO_2_/H_2_ system. Owing to the extremely low H_2_ uptake, only near-zero-coverage *Q*_st_ values are reported for H_2_. The superior CO_2_ affinity of TUS-621 can be attributed to oxygen-rich pore environments that generate stronger quadrupole–dipole interactions with CO_2_ molecules,^64^ effectively stabilizing adsorbed CO_2_ within the channels. In contrast, although sulfur atoms in TUS-622 enhance framework polarizability, their softer Lewis basicity and lower electronegativity result in weaker localized electrostatic interactions with CO_2_, leading to reduced uptake despite comparable pore sizes. Finally, the intrinsic adsorption selectivity of both frameworks toward relevant gas pairs was quantitatively evaluated using Ideal Adsorbed Solution Theory (IAST) based on experimentally measured single-component adsorption isotherms. As shown in Figure S16, both TUS-621 and TUS-622 exhibit pronounced CO_2_/CH_4_ adsorption selectivity across the investigated pressure range, with TUS-621 consistently outperforming TUS-622 due to stronger CO_2_–framework interactions coupled with suppressed CH_4_ adsorption. A similar trend is observed for CO_2_/H_2_ separation (Figure S17), where both frameworks display high selectivity because of the negligible adsorption of H_2_, and TUS-621 again performs more favorably. The pressure dependence of the selectivity curves indicates that adsorption selectivity is dominated by enthalpic interactions at low pressures, where framework–CO_2_ affinity plays a decisive role rather than pore-filling effects. Overall, the IAST analysis is consistent with the experimentally observed adsorption trends and supports the subsequent membrane-performance analysis.

**Figure 2:**
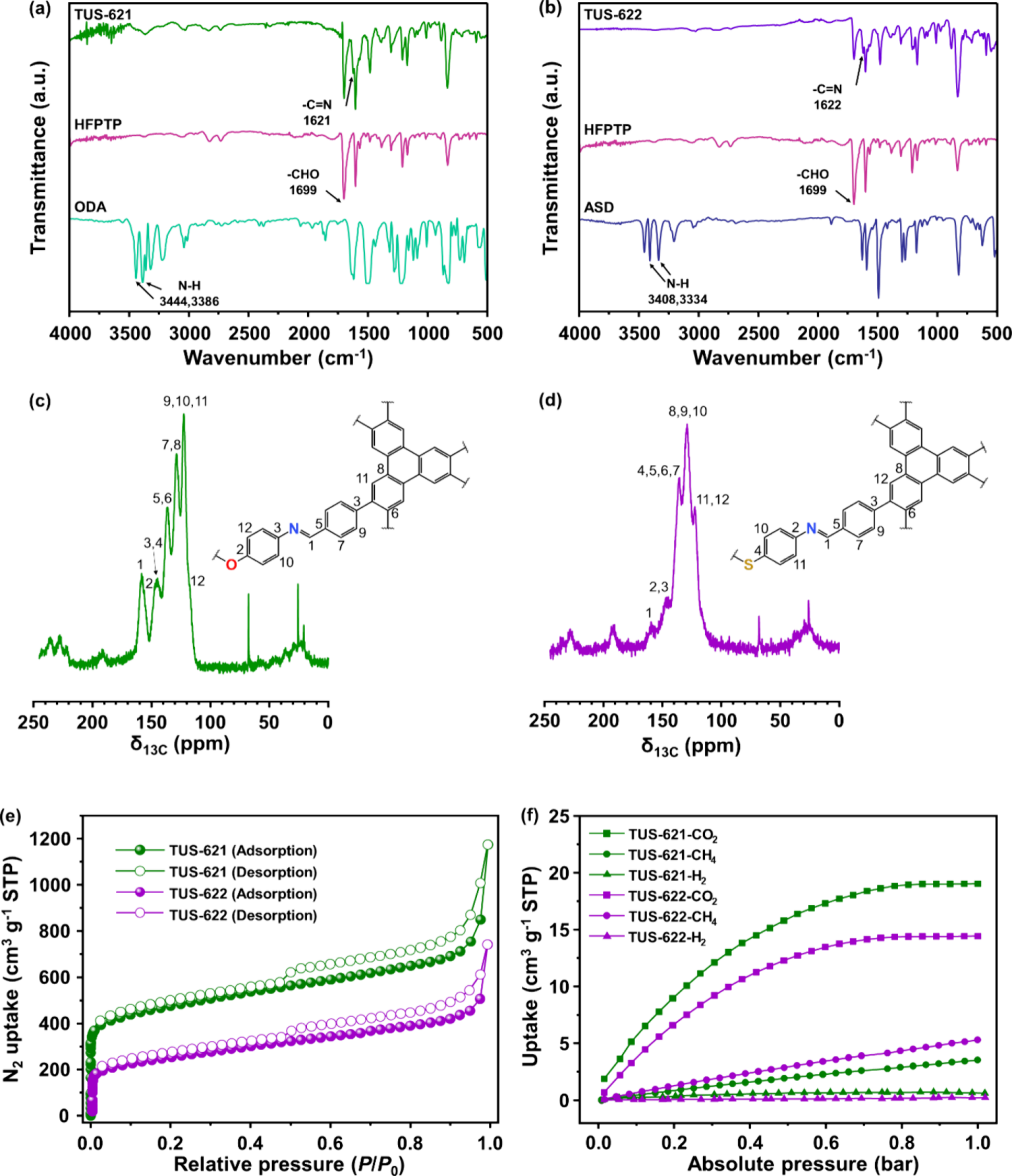
**Spectroscopic identification, porosity, and gas sorption properties of TUS-621 and TUS-622**. (a,b) FT-IR spectra of TUS-621 (a) and TUS-622 (b), confirming imine linkage formation through the appearance of C=N stretching bands and the disappearance of amine and aldehyde vibrations. (c,d) Solid-state ^13^C CP/MAS NMR spectra of TUS-621 (c) and TUS-622 (d), showing characteristic imine carbon resonances at ∼159 ppm. (e) N_2_ adsorption–desorption isotherms measured at 77 K for TUS-621 and TUS-622, demonstrating permanent microporosity with modest hysteresis. (f) Single-component CO_2_, CH_4_, and H_2_ adsorption isotherms of TUS-621 and TUS-622 at 298 K, highlighting preferential CO_2_ uptake.

### Morphological Uniformity and Thermal–Chemical Robustness of the COF Frameworks

Electron microscopy analyses provide important insights into the morphological integrity and processability of the COF materials. Scanning electron microscopy (SEM) reveals that TUS-621 crystallizes as lamellar crystallites (Figure S7), whereas TUS-622 consists of rounded to ellipsoidal particles (Figure S8). These distinct yet well-formed particle morphologies indicate controlled nucleation and growth during solvothermal synthesis, consistent with symmetry-guided framework assembly. Such morphological characteristics are particularly advantageous for MMM fabrication, as they facilitate dispersion within polymer matrices and help minimize interfacial defects that can otherwise compromise gas separation performance. The intrinsic robustness of the COF backbones was further evaluated by thermogravimetric analysis (TGA) under an inert atmosphere. TUS-621 exhibits negligible mass loss up to approximately 475 °C, while TUS-622 remains thermally stable up to approximately 430 °C (Figures S11 and S12), underscoring the exceptional thermal resilience of both frameworks. This high decomposition threshold reflects the combined effects of dense covalent connectivity, rigid aromatic building blocks, and extended π-conjugation, which collectively suppress bond cleavage and backbone rearrangement at elevated temperatures. The slightly lower thermal stability of TUS-622 relative to TUS-621 can be attributed to the incorporation of sulfide linkages, which possess lower bond dissociation energies than ether linkages but remain sufficiently robust for membrane processing and operation under demanding conditions. In addition to thermal stability, the chemical durability of the frameworks was systematically assessed through solvent-resistance tests. Upon immersion in a range of common organic solvents as well as strongly acidic and strongly alkaline aqueous media (HCl, pH = 1; NaOH, pH = 14) for 24 h, both TUS-621 and TUS-622 retained their characteristic PXRD patterns without detectable peak broadening or significant intensity loss (Figures S13 and S14), confirming preservation of their long-range crystallinity and framework integrity. This resistance to solvent-induced degradation highlights the strong imine linkages reinforced by conjugated aromatic domains and π–π stacking interactions, which together impart structural rigidity against swelling or dissolution. The combination of morphological uniformity, high thermal endurance, and outstanding chemical stability establishes TUS-621 and TUS-622 as mechanically and chemically robust COF platforms well suited for incorporation into MMMs and for sustained operation under realistic gas separation conditions.

### Fabrication, Interfacial Structure, and Gas Separation Performance of COF–Pebax MMMs

COF-based MMMs were fabricated via a solution-casting strategy in which TUS-621 or TUS-622 was homogeneously dispersed within a Pebax continuous phase to form flexible, free-standing films with controlled filler loadings. Pebax was deliberately selected as the polymer matrix owing to its well-established phase-separated architecture comprising rigid polyamide domains and flexible polyether segments, which confers an optimal balance between mechanical robustness, processability, and intrinsic CO_2_ affinity.^65^ The ether-rich soft segments of Pebax provide favorable solubility for CO_2_, while the semicrystalline hard domains impart dimensional stability, making Pebax an ideal host matrix for assessing the transport-enhancing role of porous fillers. Within this architecture, TUS-621 and TUS-622 act as dispersed molecular sieving and CO_2_-philic domains, introducing permanent porosity, well-defined transport pathways, and preferential adsorption sites that complement the solution–diffusion behavior of the polymer. Digital photographs of the membranes (Figure 3a–h, insets) reveal a progressive and uniform color evolution with increasing COF loading, indicative of successful filler incorporation and homogeneous dispersion. Neat Pebax appears light gray, whereas TUS-621/Pebax membranes gradually develop a warm yellow to wooden-yellow hue at 15 wt % loading, reflecting the intrinsic chromophoric nature of the π-conjugated COF. In contrast, TUS-622/Pebax membranes exhibit a paler yellow coloration even at the highest loading, consistent with the weaker visible absorption of the sulfur-containing framework. Importantly, the absence of visible aggregation or phase separation suggests strong interfacial compatibility between the COFs and Pebax, likely mediated by hydrogen bonding and dipole–dipole interactions between the imine/heteroatom-rich COF surfaces and the ether and amide functionalities of the polymer. SEM provides further insight into the membrane microstructure. Top-view SEM images (Figure 3a–h) show a smooth and featureless surface for neat Pebax, while MMMs display uniformly distributed COF particles embedded within the polymer matrix as the filler content increases, without evidence of interfacial voids or filler agglomeration, owing to the fully organic composition of the COF materials that affords excellent compatibility with the polymer matrix. Cross-sectional SEM images (Figure 3i–p) confirm dense, defect-free membrane interiors and reveal thicknesses of 61 ± 3.5 μm for Pebax, 53 ± 2.6, 47 ± 1.7, and 51 ± 2.1 μm for TUS-621/Pebax membranes at 5, 10, and 15 wt %, respectively, and 59 ± 3.3, 52 ± 2.0, and 55 ± 2.8 μm for the corresponding TUS-622/Pebax membranes (Table S2). These modest variations reflect changes in casting viscosity and filler content rather than structural collapse, and permeability values were therefore normalized by thickness to ensure accurate comparison. The combined SEM/EDS (Figures S18–S21) and TEM/EDS (Figures S22, S23) analyses confirm that the COF fillers are homogeneously distributed throughout the Pebax matrix at 10 wt % loading, without obvious large-scale aggregation or interfacial void formation. The structural integrity of the COFs within the polymer matrix was examined by XRD and FT-IR spectroscopy. The absence of pronounced COF diffraction peaks in the XRD patterns of the MMMs (Figures S24, S25) is attributed to the relatively low filler loadings, the dominant amorphous scattering from Pebax, and partial exfoliation or random orientation of the 2D COF crystallites within the polymer matrix—features that are commonly observed in well-dispersed MMMs and are indicative of intimate polymer–filler mixing rather than framework degradation.^66^ Consistent with this interpretation, weak features near 3.69°, corresponding to the COF (100) reflection, are observed at 15 wt % loading for both TUS-621- and TUS-622-based MMMs. FT-IR spectra further confirm the presence of COFs within the membranes, with progressively intensified imine (C=N) and C–O–C bands for TUS-621/Pebax (Figure S26) and imine and C–S bands for TUS-622/Pebax (Figure S27) as the filler content increases, while no new bands indicative of chemical degradation are observed. To further probe the interfacial interactions between the COF fillers and the Pebax matrix, the mechanical properties of the membranes were evaluated by tensile testing. As shown in Figures S28 and S29 and Table S3, incorporation of TUS-621 or TUS-622 leads to a pronounced increase in membrane stiffness at low-to-moderate filler loadings. The Young’s modulus increases from 2.96 MPa for neat Pebax to 6.69 MPa for TUS-621/Pebax-10% and 7.07 MPa for TUS-622/Pebax-10%, indicating efficient stress transfer across the filler–polymer interface and thereby supporting the presence of favorable interfacial adhesion between the COF domains and the Pebax matrix. At a higher loading of 15 wt %, the modulus decreases for both systems, suggesting that excessive filler incorporation partially disrupts the optimal polymer–filler interfacial organization, likely due to the onset of local aggregation. These mechanical results are consistent with the gas separation trends and further support that 10 wt % represents the optimal loading regime for achieving strong interfacial coupling and high membrane performance.

**Figure 3:**
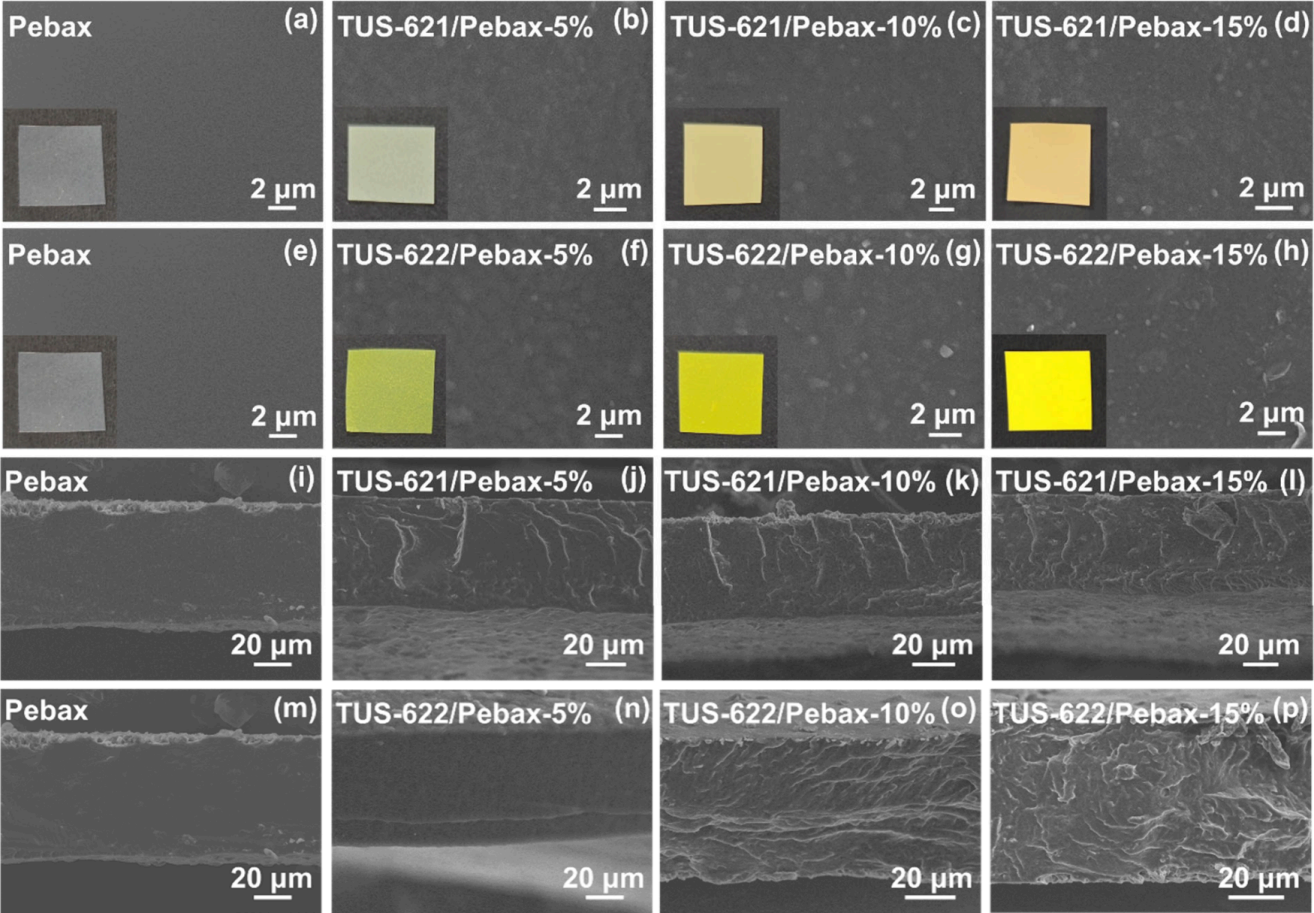
**Morphology, interfacial structure, and physical characteristics of COF–Pebax MMMs**. (a–h) Top-view SEM images of neat Pebax and TUS-621/Pebax and TUS-622/Pebax MMMs with COF loadings of 5, 10, and 15 wt %; insets show digital photographs of the corresponding free-standing membranes, illustrating uniform color evolution with increasing COF content. (i–p) Cross-sectional SEM images of neat Pebax and TUS-621/Pebax and TUS-622/Pebax MMMs at varying filler loadings, confirming dense, defect-free membrane interiors and uniform thicknesses.

Single-gas permeation measurements at 2 bar and 25 °C demonstrate that incorporation of both COFs markedly enhances CO_2_ permeability and selectivity relative to neat Pebax. For CO_2_/CH_4_ separation, the CO_2_ permeability increases from 125.0 ± 8.6 Barrer for Pebax to 428.9 ± 11.2 Barrer for TUS-621/Pebax-10% and 478.5 ± 27.0 Barrer for TUS-621/Pebax-15%, accompanied by a substantial rise in CO_2_/CH_4_ selectivity from 12.5 to a maximum of 49.87 at 10 wt % loading (Figure 4a, Table S4). A similar but more moderate enhancement is observed for TUS-622/Pebax membranes, reflecting the lower intrinsic CO_2_ affinity and accessible surface area of TUS-622. Notably, at 15 wt % loading, selectivity decreases slightly for both systems despite continued permeability gains, suggesting the onset of nonselective transport pathways arising from filler–filler proximity or local polymer chain disruption. An analogous trend is observed for CO_2_/H_2_ separation, where TUS-621/Pebax-10% again achieves the optimal balance between CO_2_ permeability (428.9 ± 11.2 Barrer) and selectivity (24.86) (Figure 4b, Table S4). Crucially, these trends are preserved under mixed-gas conditions, underscoring the intrinsic separation capability of the membranes. In binary CO_2_/CH_4_ (1:1) mixtures, TUS-621/Pebax-10% delivers a CO_2_ permeability of 433 ± 13.1 Barrer and a CO_2_/CH_4_ selectivity of 55.3 ± 2.6, closely mirroring the single-gas performance and confirming that competitive adsorption does not erode selectivity (Figure 4c, Table 1). Similarly, for CO_2_/H_2_ mixtures, TUS-621/Pebax-10% exhibits a CO_2_ permeability of 407 ± 16.7 Barrer and a selectivity of 25.2 ± 1.5 (Figure 4d, Table 1), again in excellent agreement with single-gas measurements. The consistent correspondence between single-gas and mixed-gas results indicates that separation is governed by intrinsic solubility–diffusivity coupling rather than by idealized transport assumptions or measurement artifacts.

**Figure 4:**
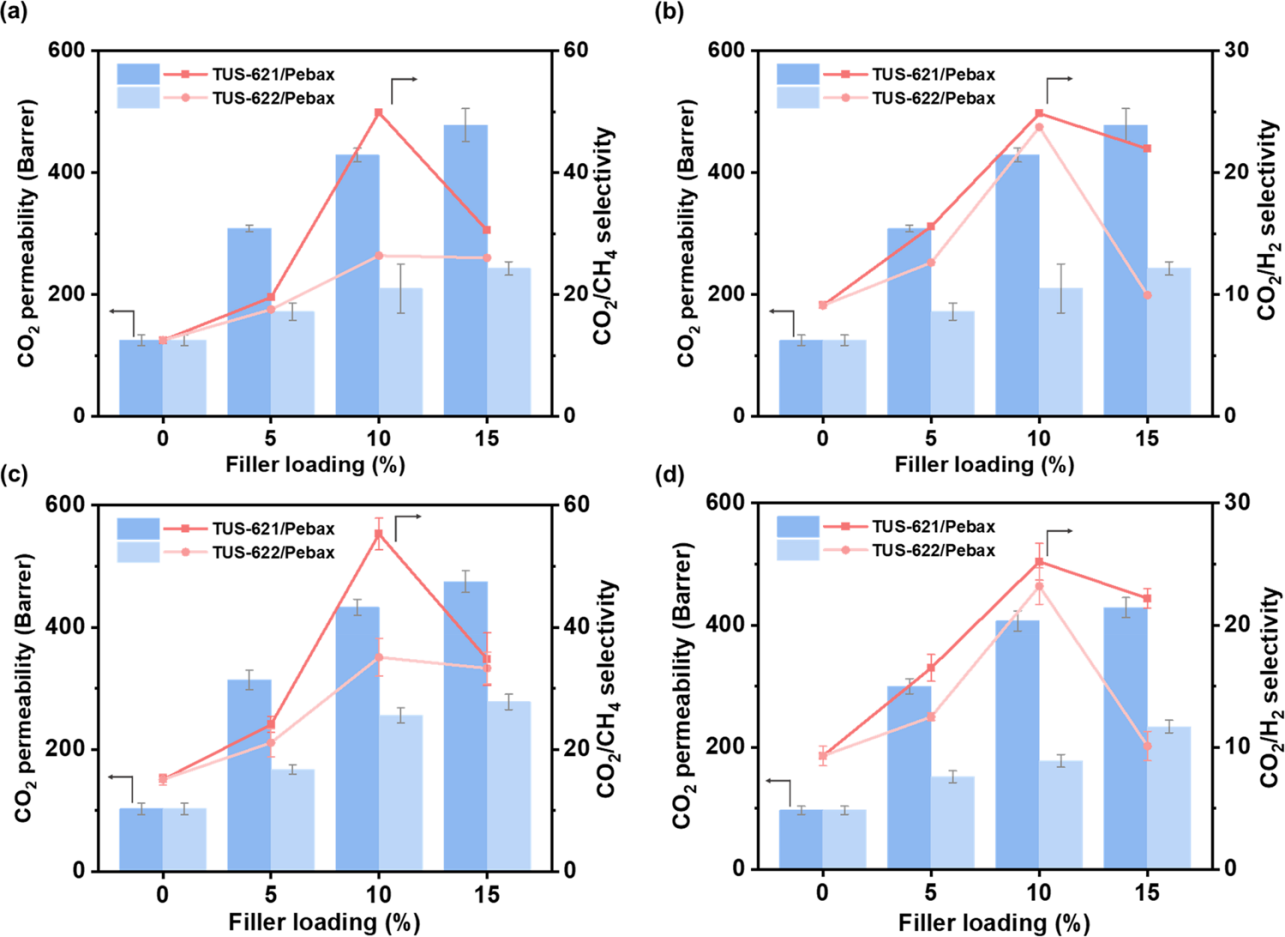
**Single-gas and mixed-gas separation performance of COF–Pebax MMMs**. (a,b) Single-gas CO_2_ permeability and ideal selectivity for CO_2_/CH_4_ (a) and CO_2_/H_2_ (b) of neat Pebax and TUS-621/Pebax and TUS-622/Pebax MMMs with varying COF loadings, measured at 2 bar and 25 °C. (c,d) Mixed-gas permeation performance for equimolar CO_2_/CH_4_ (c) and CO_2_/H_2_ (d) mixtures at 2 bar and 25 °C, showing enhanced CO_2_ permeability and selectivity upon COF incorporation.

**Table 1:** Mixed-Gas Permeation Properties of Pristine Pebax and TUS-621-and TUS-622-Based MMMs Measured at 2 bar and 25 °C

Membrane	CO_2_ permeability (Barrer)^a^	CO_2_/CH_4_ selectivity	CO_2_ permeability (Barrer)^a^	CO_2_/H_2_ selectivity
Pebax	103 ± 9.2	15.1 ± 0.9	97 ± 6.7	9.3 ± 0.8
TUS-621/Pebax-5%	314 ± 15.9	24.1 ± 1.3	300 ± 12.6	16.5 ± 1.1
TUS-621/Pebax-10%	433 ± 13.1	55.3 ± 2.6	407 ± 16.7	25.2 ± 1.5
TUS-621/Pebax-15%	475 ± 18	34.8 ± 4.3	429 ± 16.2	22.2 ± 0.8
TUS-622/Pebax-5%	167 ± 8	21.1 ± 2.3	152 ± 9.8	12.5 ± 0.3
TUS-622/Pebax-10%	256 ± 12.2	35.1 ± 3.1	178 ± 10.3	23.2 ± 1.5
TUS-622/Pebax-15%	278 ± 12.6	33.3 ± 2.6	234 ± 8.2	10.1 ± 1.2

a Permeability values were calculated by multiplying the measured gas permeance by the corresponding membrane thickness. 1 Barrer = 3.347 × 10^–16^ mol·m^–1^·s^–1^·Pa^–1^

Mixed-gas behavior is additionally influenced by competitive sorption, particularly in the CO_2_/CH_4_ system. Because TUS-621 shows stronger CO_2_ affinity whereas TUS-622 shows stronger CH_4_ affinity, CH_4_ is expected to compete more effectively against CO_2_ in TUS-622-based MMMs, reducing the relative CO_2_ sorption advantage under mixed-gas conditions. By contrast, because H_2_ adsorption is negligible, competitive adsorption effects are minimal in the CO_2_/H_2_ system. Thus, the heteroatom effect persists under mixed-gas operation because it is reflected in the competitive sorption behavior of the COF domains within the cooperative Pebax–COF transport framework. Overall, the superior performance of TUS-621/Pebax-10% and TUS-622/Pebax-10% MMMs arises from an optimal balance between enhanced CO_2_ sorption within the COF pores, efficient interfacial transport between filler and polymer, and preservation of membrane integrity. Excessive filler loading, while increasing permeability, compromises selectivity by introducing less discriminating transport pathways. These results establish 10 wt % COF loading as the optimal regime and demonstrate that heteroatom-engineered COFs can be effectively integrated into polymer matrices to achieve ultraselective CO_2_ separation under both single-gas and realistic mixed-gas conditions. To further examine the origin of the membrane-performance differences, the gas transport behavior of pristine Pebax, TUS-621/Pebax-10%, and TUS-622/Pebax-10% was analyzed using the solution–diffusion model by separating the permeability into solubility and diffusivity contributions (Tables S5, S6). For both CO_2_/CH_4_ and CO_2_/H_2_ separations, incorporation of the COF fillers increases the CO_2_ solubility coefficient relative to neat Pebax, consistent with the introduction of additional CO_2_-philic adsorption domains within the membrane. Notably, TUS-621/Pebax-10% exhibits a higher CO_2_ solubility coefficient than TUS-622/Pebax-10%, in line with the stronger CO_2_ affinity of the oxygen-lined framework indicated by the adsorption isotherms and near-zero-coverage *Q*_st_ values. At the same time, the CO_2_ diffusivity coefficient is also enhanced in the MMMs, with TUS-621/Pebax-10% again outperforming TUS-622/Pebax-10%, indicating that the performance advantage of TUS-621 cannot be assigned solely to heteroatom-dependent sorption strength. Rather, the superior separation behavior arises from the coupled effects of stronger CO_2_ affinity, greater accessible porosity and transport accessibility, and favorable framework–polymer synergy. Thus, while the controlled isostructural comparison supports a meaningful role of pore-surface heteroatom chemistry, the present results indicate that chemical and porosity-related effects are intertwined in determining the overall membrane performance.

To examine whether the observed heteroatom-dependent separation trends are specific to Pebax or reflect a broader filler design effect, additional mixed-matrix membranes were prepared using two chemically distinct polymer matrices, namely PIM-1 and polyimide (PI), and their mixed-gas CO_2_/CH_4_ separation performance was evaluated (Table S9). In all three polymer platforms—Pebax, PIM-1, and PI—incorporation of TUS-621 or TUS-622 enhances both CO_2_ permeability and CO_2_/CH_4_ selectivity relative to the corresponding pristine polymer, while TUS-621 consistently outperforms TUS-622. These cross-matrix results indicate that the beneficial influence of the heteroatom-engineered COFs is not restricted to the intrinsically CO_2_-philic Pebax matrix, but can also be expressed in a high-free-volume microporous polymer and in a glassy polymer. At the same time, the absolute magnitude of the enhancement remains matrix-dependent, indicating that the final membrane performance arises from the interplay between the intrinsic transport characteristics of the host polymer and the CO_2_-philic, molecularly defined transport domains introduced by the COF fillers. Thus, the present results support a general role for heteroatom-engineered COFs as transferable filler-level design elements, while also underscoring that the detailed transport response is governed by polymer–filler synergy rather than by filler chemistry alone.

To further examine postfabrication filler accessibility and polymer–filler compatibility under thin selective-layer conditions, thin-film nanocomposite (TFN) membranes based on TUS-621/Pebax-10% and TUS-622/Pebax-10% were also prepared and evaluated. Top-view and cross-sectional SEM images (Figures S30, S31) show continuous selective layers with thicknesses of approximately 560 and 450 nm for the TUS-621- and TUS-622-based TFN membranes, respectively. Despite their small thickness, both TFN membranes retain clear mixed-gas CO_2_ separation capability, delivering CO_2_/CH_4_ selectivities of 33.5 and 28.2 and CO_2_/H_2_ selectivities of 21.4 and 18.5, respectively (Tables S10, S11). These results further support good polymer–filler compatibility and indicate that the COF-containing selective layers remain continuous and functionally selective after thin-film fabrication.

### Operational Stability, Pressure–Temperature Dependence, and Benchmarking against the Robeson Upper Bound

Mixed-gas permeation measurements under variable pressure, temperature, and prolonged operation were conducted to rigorously assess the robustness, practical relevance, and intrinsic transport mechanisms of the optimized TUS-621/Pebax-10% and TUS-622/Pebax-10% MMMs under conditions representative of industrial gas separation. Evaluating performance over a pressure range of 2–10 bar is particularly important for CO_2_ separations associated with natural gas upgrading and hydrogen purification, where membranes are exposed to elevated trans-membrane pressures that can induce polymer chain compaction, filler–polymer interfacial stress, or nonideal competitive adsorption effects. As shown in Figure 5a, both MMMs exhibit high and pressure-stable CO_2_/CH_4_ separation performance at 25 °C. With increasing pressure, a slight decrease in CO_2_ permeability is observed for both membranes, which can be attributed to gradual polymer chain densification and partial saturation of CO_2_ sorption sites within the Pebax matrix and COF pores. Importantly, the CO_2_/CH_4_ selectivity remains nearly constant across the entire pressure range, indicating that the dominant separation mechanism—preferential CO_2_ sorption combined with size-selective diffusion—remains intact and is not compromised by competitive adsorption or pressure-induced nonselective transport. TUS-621/Pebax-10% consistently displays higher permeability and selectivity than TUS-622/Pebax-10%, consistent with its stronger CO_2_ affinity, higher effective CO_2_ solubility, and greater accessible porosity. A similar pressure-independent selectivity trend is observed for CO_2_/H_2_ separation (Figure 5b). Although CO_2_ permeability decreases modestly with increasing pressure, CO_2_/H_2_ selectivity remains essentially unchanged for both MMMs. This behavior arises because H_2_ exhibits negligible adsorption and diffuses primarily through the polymer matrix, while CO_2_ transport is governed by selective sorption within the COF domains and ether-rich Pebax segments. The stability of selectivity under pressure confirms that transport is dominated by intrinsic solubility–diffusivity coupling rather than pressure-driven competitive effects, underscoring the reliability of these membranes under realistic operating pressures.

**Figure 5:**
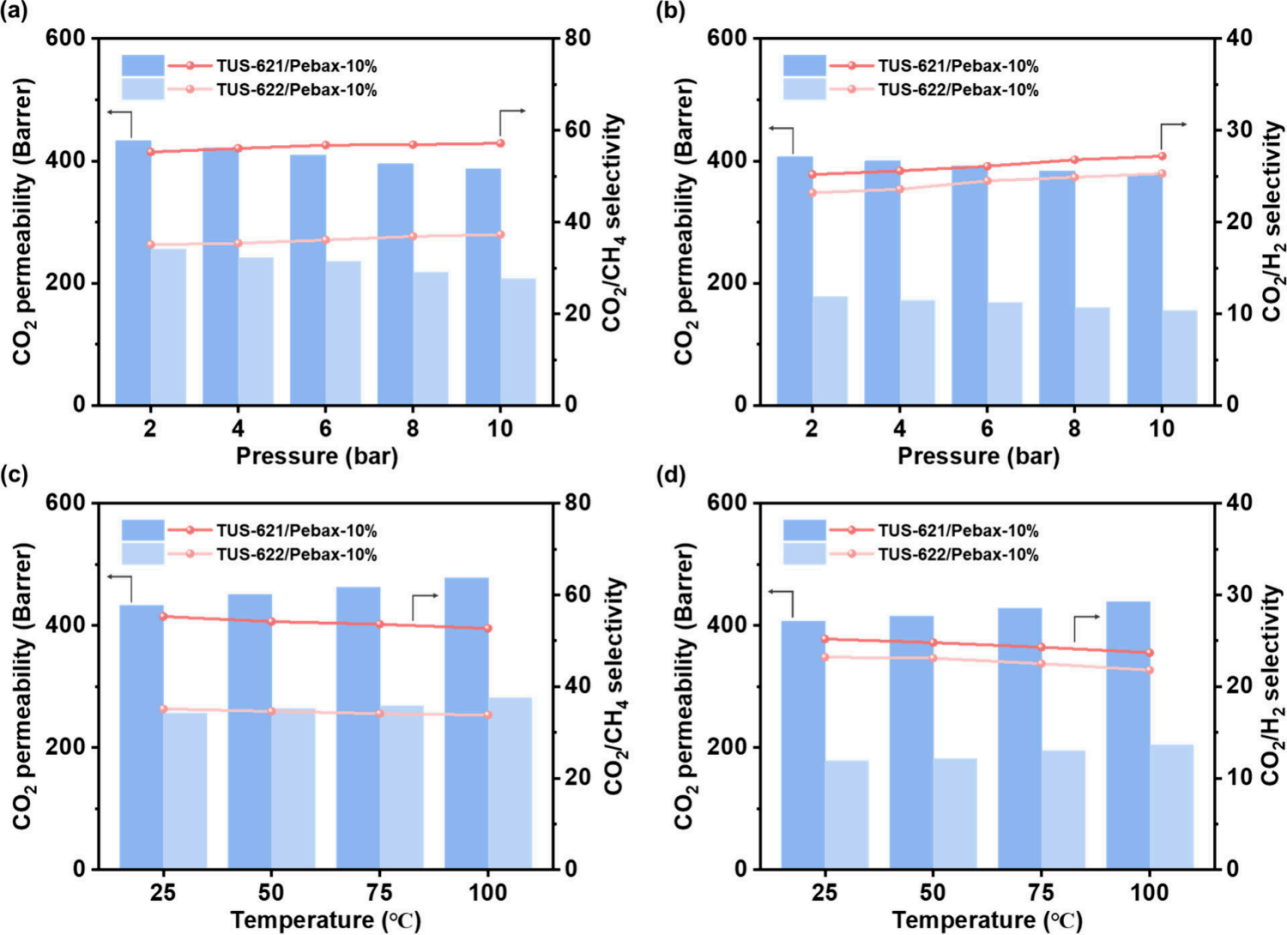
**Pressure- and temperature-dependent mixed-gas separation performance of optimized COF–Pebax MMMs**. (a,b) CO_2_ permeability and separation selectivity for CO_2_/CH_4_ (a) and CO_2_/H_2_ (b) of TUS-621/Pebax-10% and TUS-622/Pebax-10% membranes measured under mixed-gas conditions at 25 °C over a pressure range of 2–10 bar. (c,d) CO_2_ permeability and selectivity for CO_2_/CH_4_ (c) and CO_2_/H_2_ (d) as a function of temperature (25–100 °C) at 2 bar, demonstrating stable selectivity and thermally activated CO_2_ transport.

To further evaluate thermal robustness, mixed-gas permeation experiments were conducted from 25 to 100 °C at 2 bar. This temperature range probes the balance between thermally activated diffusion and the exothermic nature of CO_2_ adsorption. As shown in Figures 5c and 5d, CO_2_ permeability increases gradually with temperature for both MMMs, consistent with enhanced polymer segmental mobility and accelerated gas diffusion. In contrast, CO_2_/CH_4_ and CO_2_/H_2_ selectivities either remain nearly constant or decrease slightly with temperature. This modest decline originates from reduced CO_2_ adsorption enthalpy at elevated temperatures and a relatively stronger increase in diffusivity for CH_4_ and H_2_ compared to CO_2_.^67^ Nevertheless, even at 100 °C, both membranes retain high selectivity, demonstrating that the COF-induced transport pathways effectively suppress nonselective diffusion and preserve separation efficiency under thermally demanding conditions.

Long-term operational stability is a critical requirement for practical membrane deployment. Accordingly, continuous mixed-gas permeation tests were performed for 30 days at 2 bar and 25 °C, during which the membranes were maintained under the operating conditions throughout the entire test period, and 20 parallel data sets were collected each day to ensure reproducibility and statistical reliability. As illustrated in Figures 6a and 6b, both TUS-621/Pebax-10% and TUS-622/Pebax-10% exhibit remarkably stable CO_2_ permeability and separation selectivity for both CO_2_/CH_4_ and CO_2_/H_2_ gas pairs over the entire testing period. The absence of performance decay indicates excellent resistance to physical aging, filler agglomeration, and interfacial degradation. This stability is attributed to the rigid, π-conjugated COF backbones that resist structural collapse, as well as strong interfacial interactions between the COFs and Pebax that effectively immobilize polymer chains and suppress long-term densification.

**Figure 6:**
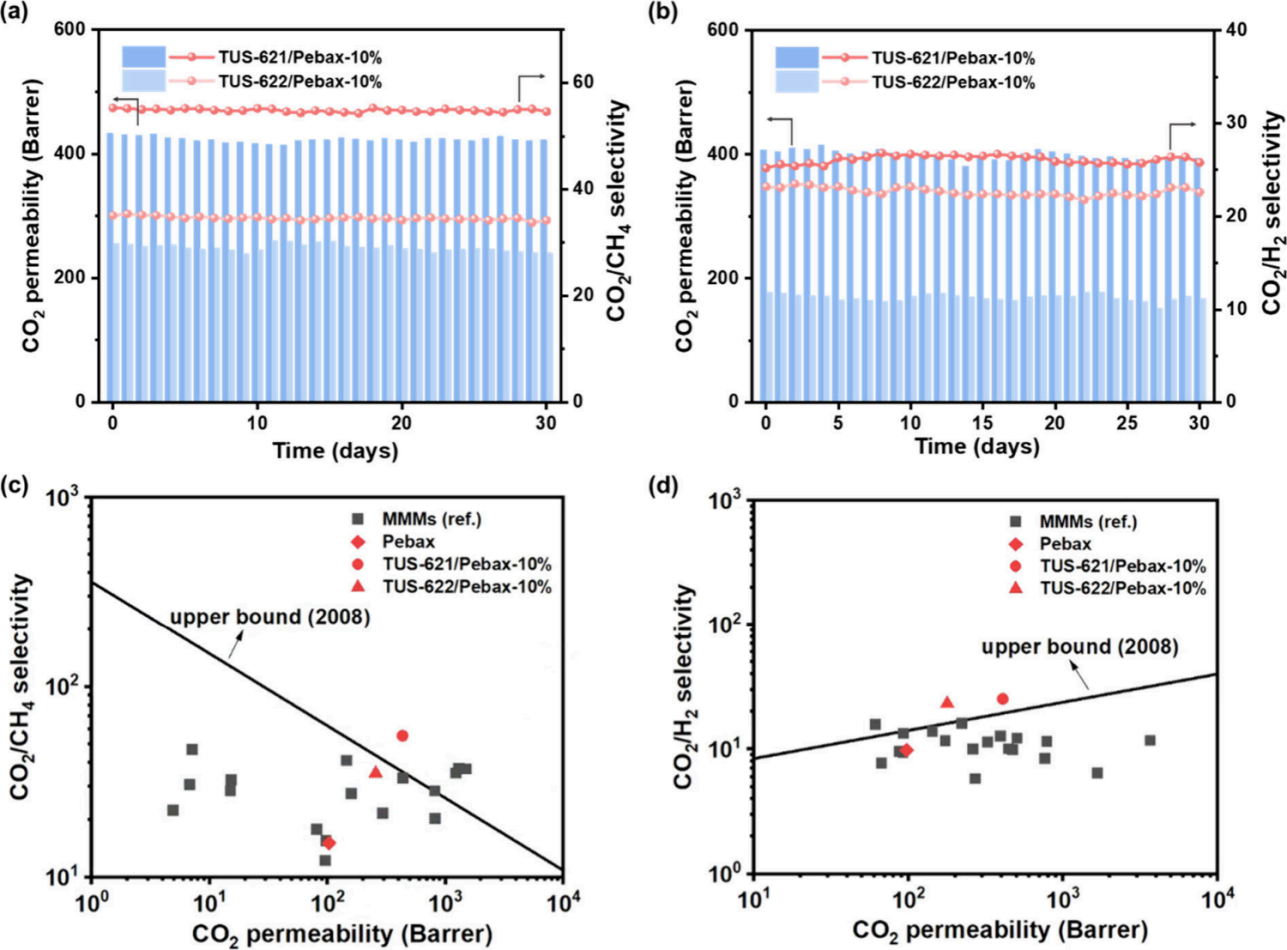
**Operational stability and benchmarking of optimized COF–Pebax MMMs**. (a,b) Long-term stability of CO_2_ permeability and separation selectivity for CO_2_/CH_4_ (a) and CO_2_/H_2_ (b) of TUS-621/Pebax-10% and TUS-622/Pebax-10% membranes measured under mixed-gas conditions at 2 bar and 25 °C over 30 days. (c) Robeson upper-bound plot (2008) for CO_2_/CH_4_ separation, comparing the performance of TUS-621/Pebax-10% and TUS-622/Pebax-10% with representative MMMs reported in the literature. (d) Robeson upper-bound plot (2008) for CO_2_/H_2_ separation, highlighting the superior permeability–selectivity combinations achieved by the COF-based MMMs.

The separation performance of the optimized MMMs was further benchmarked against the Robeson upper bound,^14,15^ which represents the empirical trade-off between permeability and selectivity for polymer-based membranes and serves as a critical metric for evaluating true material advancement. In the CO_2_/CH_4_ performance map (Figure 6c), TUS-621/Pebax-10% clearly surpasses the 2008 upper bound, achieving a CO_2_ permeability of 433 Barrer and a CO_2_/CH_4_ selectivity of 55.3, thereby outperforming most reported MMMs under comparable conditions (Table S7). In contrast, TUS-622/Pebax-10% approaches the upper bound with a permeability of 256 Barrer and selectivity of 35.1, representing a substantial improvement over neat Pebax (103 Barrer, 15.1). The superior performance of TUS-621-based MMMs arises from the higher intrinsic surface area, stronger quadrupole–dipole interactions between CO_2_ and ether-rich pore walls, and more efficient solubility-selective transport pathways relative to the sulfur-containing TUS-622 framework. An analogous trend is observed for CO_2_/H_2_ separation. As shown in Figure 6d, both TUS-621/Pebax-10% and TUS-622/Pebax-10% exceed the Robeson upper bound, achieving CO_2_ permeabilities of 407 and 178 Barrer and CO_2_/H_2_ selectivities of 25.2 and 23.2, respectively (Table S8). The dramatic enhancement relative to neat Pebax (97 Barrer, 9.3) highlights the effectiveness of COF incorporation in suppressing H_2_ transport while amplifying CO_2_ permeability. In this case, the difference between TUS-621 and TUS-622 is less pronounced than for CO_2_/CH_4_ separation, reflecting the dominant role of molecular sieving and negligible H_2_ adsorption, which diminish the influence of heteroatom chemistry. Collectively, these results demonstrate that TUS-621/Pebax-10% represents an optimal convergence of permeability, selectivity, operational stability, and robustness, achieving ultraselective CO_2_ separation under both single-gas and mixed-gas conditions, across wide pressure and temperature ranges, and over extended operation. The consistent superiority of TUS-621-based MMMs over TUS-622-based counterparts can be attributed to the combined structural and transport characteristics of the COFs, including higher accessible surface area, oxygen-rich CO_2_-philic pore environments, and favorable framework–polymer synergy. These findings establish heteroatom-engineered COFs as powerful tools for transcending traditional permeability–selectivity trade-offs and advancing MMMs beyond the Robeson upper bound for practical CO_2_ separations.

### Computational Elucidation of Gas Adsorption and Heteroatom-Dependent CO_2_ Interactions

The structures of TUS-621 and TUS-622 were modeled using single-layer periodic unit cells for all calculations, with each framework containing 165 atoms (Figure 7a,b). To understand the origin of the different gas selectivities toward CO_2_, H_2_, and CH_4_ in TUS-621 and TUS-622, we first examined their electronic structures through density of states (DOS) analysis (Figure 7d,e). Notably, both frameworks exhibit nearly identical global electronic characteristics. The overall DOS profiles as well as the *p*-orbital occupancy and *p*-band centers are essentially indistinguishable.This suggests that, from a bulk electronic perspective, the two COFs are electronically similar, despite their markedly different gas adsorption behavior. Given this similarity, we next focused on the key structural differences between the two frameworks. TUS-622 contains a sulfur atom in place of the oxygen present in TUS-621. Accordingly, we identified the oxygen site in TUS-621 and the sulfur site in TUS-622 as the respective adsorption centers and investigated their roles in competitive gas adsorption. Our calculations reveal that both COFs follow the same adsorption trend: CO_2_ > CH_4_ > H_2_, with CO_2_ exhibiting the strongest adsorption in both systems. However, TUS-621 consistently shows an adsorption strength higher than that of TUS-622 for all gases (Figure 7c). The adsorption remains physisorptive in nature because the adsorption is primarily governed by physisorption, identifying a clear electronic origin for the enhanced activity of TUS-621 is nontrivial. Nevertheless, the projected DOS analysis provides important insights. CO_2_ exhibits hybridization with the states of oxygen in TUS-621 compared to its interaction with states of sulfur in TUS-622 (Figure 7f,g). The hybridized features are highlighted by arrows in Figure 7f. The degree of hybridization between the active sites and adsorbed CO_2_ can be understood by examining the electron population associated with the CO_2_-derived states below the Fermi level (Figure 7f, g). In TUS-621, the O active site contributes 0.80, 0.80, and 0.54 electrons to peaks a, b, and c, respectively shown in Figure 7f, giving a total of 2.14 electrons involved in hybridization with CO_2_. In comparison, the S active site in TUS-622 shows much smaller contributions of 0.01, 0.14, and 1.40 electrons for the same peaks as shown in Figure 7g, resulting in a total of 1.55 electrons. Overall, this indicates that CO_2_ interacts more strongly with TUS-621, with ∼0.6 additional electrons participating in hybridized states. Moreover, the near absence of electrons in peaks a and b for TUS-622 further suggests weaker electronic interaction between CO_2_ and the S site. Furthermore, upon adsorption, CO_2_ shows peak broadening in TUS-621 relative to TUS-622 (Figure 2h, i), indicative of stronger electronic coupling between the adsorbate and framework. This enhanced hybridization and DOS broadening collectively suggest a more effective electronic interaction between CO_2_ and the oxygen site in TUS-621. It is worth noting that our analysis is based on a single repeating layer of the COFs; inclusion of additional layers could further enhance dispersion-driven physisorption interactions.^68^

**Figure 7:**
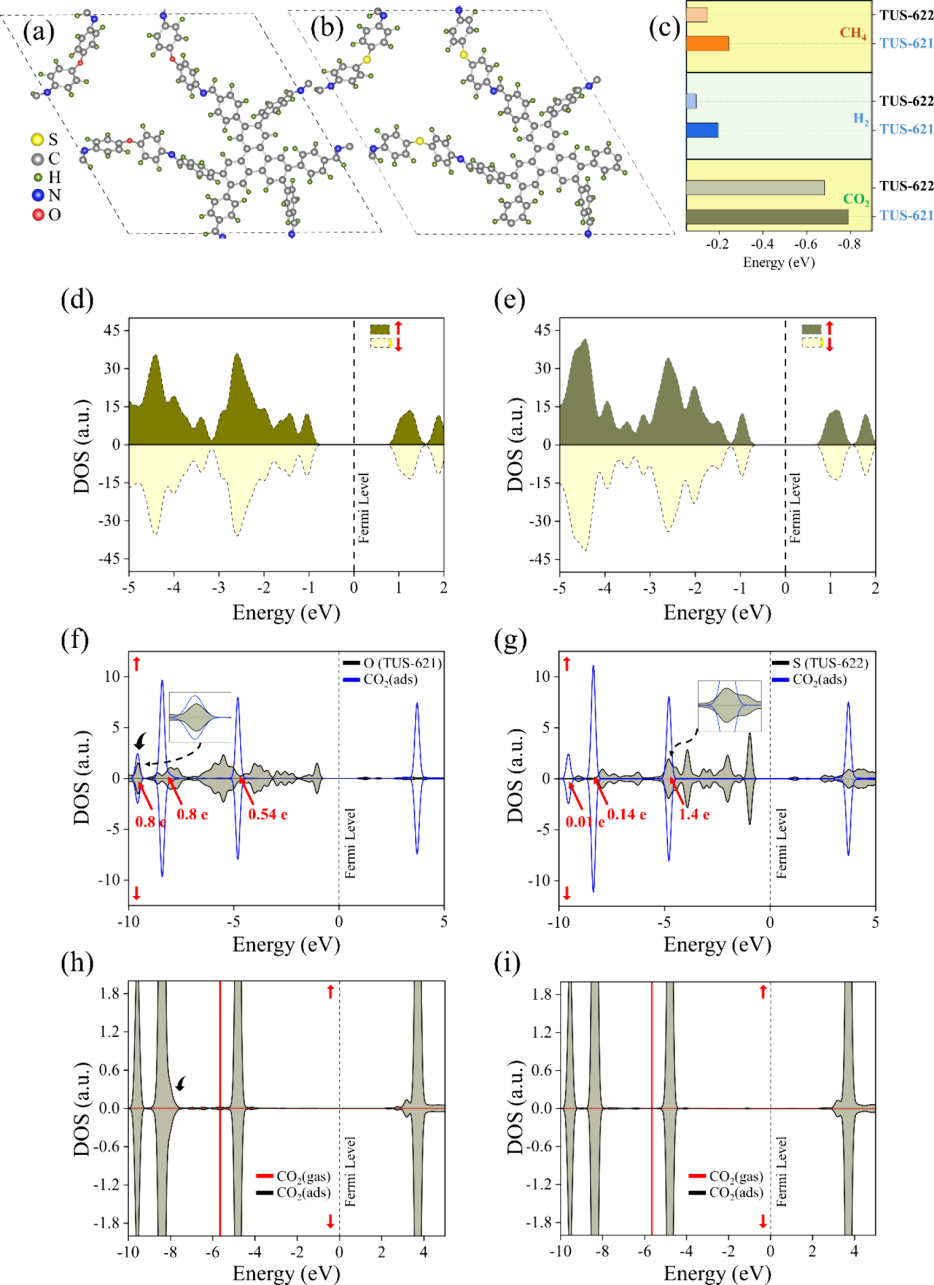
**Theoretical insight into gas adsorption and heteroatom–CO**_**2**_ **electronic interactions in TUS-621 and TUS-622**. Structural models of (a) TUS-621 and (b) TUS-622. (c) Comparative adsorption behavior of CO_2_, CH_4_, and H_2_ in TUS-621 and TUS-622. Total density of states (DOS) of (d) TUS-621 and (e) TUS-622. Projected density of states (PDOS) illustrating the interaction between adsorbed CO_2_ and the heteroatom sites: (f) O in TUS-621 and (g) S in TUS-622. Comparison of the DOS of free and adsorbed CO_2_ in (h) TUS-621 and (i) TUS-622, highlighting the electronic coupling upon adsorption.

## 3 Conclusion

In summary, this study demonstrates that deliberate heteroatom engineering within structurally precise COFs provides a powerful and generalizable route to overcoming the long-standing permeability–selectivity trade-off in membrane-based CO_2_ separations. By designing two isostructural, π-conjugated 2D COFs that differ only in their pore-lining heteroatoms, we demonstrate a clear correlation between molecular-level pore chemistry and macroscopic gas transport behavior in MMMs within this controlled isostructural COF platform. When embedded within a Pebax matrix, both COFs act as permanently porous, CO_2_-philic transport domains that synergistically enhance CO_2_ solubility and diffusion while preserving membrane integrity and processability. Among the two systems, the oxygen-rich framework TUS-621 emerges as particularly effective, delivering a rare combination of high CO_2_ permeability, exceptional CO_2_/CH_4_ and CO_2_/H_2_ selectivity, and remarkable operational stability under mixed-gas conditions, elevated pressures, broad temperature ranges, and prolonged operation. The ability of TUS-621-based MMMs to decisively surpass the Robeson upper bound underscores that the observed performance gains are substantial and appear to arise from a cooperative transport regime shaped by heteroatom-tailored pore environments, accessible porosity, and strong framework–polymer synergy. Importantly, the close agreement between single-gas and mixed-gas permeation results confirms that separation is governed by intrinsic solubility–diffusivity coupling rather than idealized or transient effects. Beyond the specific materials reported here, this work establishes a clear design principle for next-generation separation membranes: precise control over COF pore chemistry—rather than topology alone—can be leveraged to rationally tune gas–framework interactions and break conventional transport limits. More broadly, heteroatom-engineered COFs offer a versatile and modular platform for advancing MMMs toward practical, energy-efficient CO_2_ separations relevant to natural gas upgrading, hydrogen purification, and carbon management technologies.

## Supplementary Material



## Notes

Materials and methods, nitrogen sorption, SEM, TEM, TGA, chemical stability analysis, isosteric heat of adsorption (*Q*_st_), IAST-based selectivity calculations, membrane thickness, XRD patterns of MMMs, FT-IR spectra of membranes, tensile test of MMMs, single-gas permeation performance, diffusivity and solubility coefficients of Pebax and MMMs, comparison of gas separation performance with previously reported membranes, CO_2_/CH_4_ mixture gas separation performance of COF/PIM-1 and COF/PI MMMs, SEM characterization and mixed-gas separation performance of TUS-621/Pebax-10% and TUS-622/Pebax-10% TFN membranes, and unit cell information and fractional atomic coordinates (PDF)
