# Supplementary material for: Heteroatom-Engineered Covalent Organic Frameworks Break the CO2 Separation Trade-Off in Mixed Matrix Membranes
Source: J Am Chem Soc. 2026 May 21;148(21):21406–19. doi: 10.1021/jacs.5c23169 (PMC13244459; doi:10.1021/jacs.5c23169)
Supplement: Supplementary file 1 [file ja5c23169_si_001.pdf]

Supporting Information for:

Heteroatom-Engineered Covalent Organic Frameworks Break the CO_2 Separation Trade-Off in Mixed Matrix Membranes

Tsukasa Irie,[a,†] Liting Yu,[b,†] Sourav Ghosh,[c] Mika Nozaki,[a,] Kohki Sasaki,[a] Tokuhisa Kawawaki,[a] Ranjit Thapa,[c,d]* Yu Zhao,*[e] Saikat Das,*[a] Zixi Kang,*[b,f] and Yuichi Negishi*[a]

[a]Institute of Multidisciplinary Research for Advanced Materials, Tohoku University, 2-1-1 Katahira, Aoba-ku, Sendai 980-8577, Japan
[b]Shandong Key Laboratory of Intelligent Energy Materials, School of Materials Science and Engineering, China University of Petroleum (East China), Qingdao, Shandong, 266580, PR China
[c]Department of Physics, SRM University–AP, Amaravati 522 240, Andhra Pradesh, India
[d]Centre for Computational and Integrative Sciences, SRM University–AP, Amaravati 522 240, Andhra Pradesh, India
[e]Zhejiang Engineering Laboratory for Green Syntheses and Applications of Fluorine-Containing Specialty Chemicals, Institute of Advanced Fluorine-Containing Materials, Zhejiang Normal University, 321004 Jinhua, China
[f]State Key Laboratory of Heavy Oil Processing, China University of Petroleum (East China), Qingdao, Shandong, 266580, PR China
[†]These authors contributed equally

*Corresponding Author
R.T.: ranjit.t@srmap.edu.in
Y.Z.: zhaoyu@zjnu.edu.cn
S.D.: das.saikat.c4@tohoku.ac.jp
Z.K.: kzx@upc.edu.cn
Y.N.: yuichi.negishi.a8@tohoku.ac.jp

List of Contents

1. Materials and Methods S3

2. Nitrogen sorption S11

3. Scanning electron microscopy (SEM) S18

4. Transmission electron microscopy (TEM) S19

5. Thermogravimetric analysis (TGA) S20

6. Chemical stability analysis S21

7. Q_{st} of TUS-621 and TUS-622 S22

8. Ideal Adsorbed Solution Theory (IAST)–based selectivity calculations S23

9. Membrane thickness S24

10. SEM images and mapping (top-view and cross-section) S25

11. TEM of TUS-621/Pebax-10% and TUS-622/Pebax-10% S26

12. XRD patterns of MMMs S27

13. FT-IR spectra of membranes S28

14. Tensile test of Pebax and MMMs S29

15. Single-gas permeation performance S31

16. D and S of Pebax and MMMs S32

17. Comparison of gas separation performance with previously reported membranes S33

18. CO_2/CH_4 mixture gas separation performance of COF/PIM-1 and COF/PI MMMs S35

19. SEM characterization and mixed-gas separation performance of TUS-621/Pebax-10% and TUS-622/Pebax-10% TFN membranes S36

20. Unit cell information and fractional atomic coordinates S37

21. Supplementary references S41

1. Materials and Methods

1.1. Chemicals. All reagents and solvents were of analytical grade. Anhydrous mesitylene, 1,4-dioxane, acetic acid (AcOH), tetrahydrofuran (THF), acetone, dichloromethane (DCM), methanol, and *n*-hexane were obtained from FUJIFILM Wako Pure Chemical Corporation. Pure Milli-Q water (>18 MΩ · cm) was generated using a Merck Millipore Direct 3 UV system. Ethanol (C_2H_5OH, 98%) was obtained from FUJIFILM Wako Pure Chemical Corporation and Tianjin Fuyu Fine Chemical. Pebax MH 1657 was obtained from Arkema. 2,3,5,6-tetrafluoroterephthalonitrile (TFTPN, 98%, Alfa Aesar) and 5,5',6,6'-tetrahydroxy-3,3,3',3'-tetramethyl-1,1'-spirobisindane (TTSBI, 97%, Alfa Aesar) were purified before use. Potassium carbonate (K_2CO_3, 99%, Energy Chemical), polydimethylsiloxane (PDMS, Shanghai D&B Biological Science and Technology), tetraethyl orthosilicate (TEOS, 98%, Macklin), dibutyltin dilaurate (DBTDL, 95%, Macklin), n-heptane (98%, Tianjin Fuyu Fine Chemical), methanol (MeOH, 99%, Tianjin Fuyu Fine Chemical), dichloromethane (99.5%, Tianjin Fuyu Fine Chemical), chloroform (99.5%, Tianjin Fuyu Fine Chemical), N,N-dimethylformamide (DMF, 98%, Energy Chemical), polyacrylonitrile substrate (PAN, Beijing Separate Equipment), and polyimide (Zhanyang Co., Ltd) were used as received.

1.2. Characterization.

Powder X-ray diffraction (PXRD): PXRD patterns of powdered samples were collected using a Rigaku MiniFlex X-ray diffractometer equipped with a Cu Kα source (λ = 1.5418 Å), operated at 40 kV and 15 mA. Data were acquired over a 2θ range of 3–40° with a step size of 0.01° and a scanning speed of 2.8° per minute. XRD measurements of membrane samples were performed on a Shimadzu XRD-6000 diffractometer using Cu Kα radiation, with a scanning speed of 10° min^{-1} over the same 2θ range (3–40°).

Fourier transform infrared (FT-IR) spectroscopy: FT-IR spectra of powdered samples were collected using a JASCO FT/IR-6600 spectrometer operated in attenuated total reflectance (ATR) mode, over the wavenumber range of 4000–500 cm^{-1}. FT-IR measurements of the membranes were performed on a PerkinElmer Fourier transform infrared spectrometer over the same wavenumber range.

Solid-state ^{13}C cross-polarization magic-angle-spinning (CP-MAS) NMR spectroscopy: Solid-state ^{13}C CP-MAS NMR spectra were obtained on a Bruker Biospin Avance III 600 NMR spectrometer equipped with a 4-mm probe, operating at a spinning frequency of 10 kHz.

Scanning electron microscopy (SEM): SEM images of powdered COF samples were acquired using a JEOL JSM-7800F field emission scanning electron microscope operated at an accelerating voltage of 7 kV. Prior to imaging, the samples were sputter-coated with a thin platinum layer to minimize

charging effects under the electron beam. Top-view and cross-sectional morphologies of the membrane samples were examined using a field-emission scanning electron microscope (FESEM; Hitachi Regulus 8100).

Transmission electron microscopy (TEM): High-resolution TEM (HR-TEM) images, along with the corresponding fast Fourier transform (FFT) and selected-area electron diffraction (SAED) patterns, were obtained using a JEOL JEM-2100F transmission electron microscope operated at an accelerating voltage of 80 kV.

Nitrogen sorption: Nitrogen uptake measurements were carried out at 77 K on a Quantachrome Autosorb iQ3 analyzer after vacuum activation of the COFs at 120 °C for 8 h under a turbomolecular pump. Specific surface areas were determined by multipoint Brunauer–Emmett–Teller (BET) analysis, and pore size distributions were obtained from the adsorption branch of the isotherms employing quenched solid density functional theory (QSDFT).

CO_2, CH_4 and H_2 gas sorption: Gas adsorption isotherms in the pressure range of 0–1 bar were measured using a Micromeritics ASAP 2020 surface area and pore size analyzer with ultra-high-purity CO_2, H_2 (99.999% purity), and CH_4 (99.99% purity) gases.

Thermogravimetric analysis (TGA): TGA profiles were collected using a Thermo Plus EVO2 instrument over a temperature range from ambient to 800 °C at a heating rate of 10 °C min^{-1} under a nitrogen flow rate of 50 mL min^{-1}.

Elemental analyses: Elemental analysis of the COF was determined using a Micro Corder JM10 elemental analyzer.

Gas separation measurements: The gas separation performance of the membranes was evaluated using a gas chromatograph (GC-9860-5C-NJ, Nanjing Haerpu Analytical Equipment) in conjunction with a gas permeation system (CY-3, Xuzhou North Gaori Electronic Equipment, Nanjing Tech University).

Tensile test of Pebax and mixed-matrix membranes (MMMs): The stress and strain curves were tested by the INSTRON 34SC-1 Tensile Tester.

1.3. Synthesis of building blocks. 2,3,6,7,10,11-hexakis(4-formylphenyl)triphenylene (HFPTP)[1], 4,4'-diaminodiphenyl ether (ODA)[2], and bis(4-aminophenyl) sulfide (ASD)[3] were synthesized in accordance with previously reported literature procedures.

1.4. Synthesis of TUS-621. HFPTP (18.8 mg, 0.022 mmol) and ODA (13.2 mg, 0.066 mmol) were mixed by thorough grinding and loaded into a Pyrex tube (8 mm ID, 10 mm OD). After addition of anhydrous mesitylene (0.8 mL), the suspension was sonicated for 15 min. Anhydrous 1,4-dioxane (0.2 mL) was subsequently introduced, followed by a further 15 min of sonication. Subsequently, 9 M aqueous acetic acid (0.1 mL) was added dropwise, and the mixture was sonicated for an additional 15 min. The reaction vessel was flash-frozen in liquid nitrogen (77 K), evacuated through three freeze–pump–thaw cycles, flame-sealed under vacuum, and heated at 120 °C for 72 h. After cooling to ambient temperature, the resulting solid was collected by centrifugation, repeatedly washed with THF, and Soxhlet-extracted with THF for 24 h. Final drying under vacuum at 100 °C for 8 h afforded TUS-621 as a yellow powder in 74% yield. Anal. Calcd. for $C_{96}H_{60}N_6O_3$: C: 85.69; H: 4.49; N: 6.25; O: 3.57. Found: C: 83.72; H: 4.67; N: 5.84; O: 3.75.

1.5. Synthesis of TUS-622. HFPTP (18.8 mg, 0.022 mmol) and ASD (14.3 mg, 0.066 mmol) were mixed by thorough grinding and loaded into a Pyrex tube (8 mm ID, 10 mm OD After addition of anhydrous mesitylene (1.0 mL), the suspension was sonicated for 15 min. Subsequently, 6 M aqueous acetic acid (0.1 mL) was added dropwise, and the mixture was sonicated for an additional 15 min. The reaction vessel was flash-frozen in liquid nitrogen (77 K), evacuated through three freeze–pump–thaw cycles, flame-sealed under vacuum, and heated at 120 °C for 72 h. After cooling to ambient temperature, the resulting solid was collected by centrifugation, repeatedly washed with THF, and Soxhlet-extracted with THF for 24 h. Final drying under vacuum at 100 °C for 8 h afforded TUS-622 as a yellow powder in 71% yield. Anal. Calcd. for $C_{96}H_{60}N_6S_3$: C: 82.71; H: 4.34; N: 6.03; S: 6.90. Found: C: 76.58; H: 5.13; N: 4.59; S: 7.69.

1.6. Preparation of TUS-621/Pebax and TUS-622/Pebax MMMs. COF-based MMMs were prepared by incorporating TUS-621 or TUS-622 into a Pebax polymer matrix via a solution-casting method. Briefly, a predetermined amount of TUS-621 or TUS-622 was dispersed in 6 mL of a mixed solvent consisting of ethanol and deionized water (C_2H_5OH/H_2O = 7:3, w/w). The suspension was subjected to ultrasonication followed by magnetic stirring for 8 h to ensure homogeneous dispersion of the COF particles, affording solution A. Separately, Pebax was dissolved in 6 mL of the same ethanol/water mixed solvent (7:3, w/w) under continuous stirring at 80 °C until a clear and homogeneous polymer solution was obtained (solution B). After complete dissolution, solutions A and B were combined and further stirred for more than 8 h to promote uniform mixing and interfacial compatibility between the COF fillers and the polymer matrix. The resulting casting solution was poured into a flat-bottomed glass Petri dish and allowed to undergo slow solvent evaporation at ambient temperature to form a free-standing membrane. The obtained membranes were carefully peeled from the substrate and

subsequently dried in a vacuum oven at 60 °C overnight to remove residual solvent. MMMs were prepared with COF loadings of 5, 10, and 15 wt% (relative to the total membrane mass).

1.7. Purification of TTSBI. Methanol (90 mL) was added to a conical flask containing TTSBI (13 g), and the flask was sealed, stirred, and heated at 60 °C. Stirring was stopped when the reactant color turned from brown to off-white. The flask was left open, and the heating temperature was increased to 65 °C. Stirring was continued until the solvent volume was 40 mL, and heating was stopped. After cooling to room temperature, 20 mL of dichloromethane was added to the conical flask and stirred for 0.5 h. After cooling the conical flask to -20 °C in the refrigerator, the precipitated crystals were collected by filtration and washed 3 times with methanol. The product was vacuum-dried for 24 h at 60 °C to obtain white powder TTSBI. The yield was ~40%.

1.8. Purification of TFTPN. TFTPN was placed in a micro-sublimator, and the sample was vacuum sublimated at 150 °C to collect the condensed, white product. The yield was ~95%.

1.9. Synthesis of polymer of intrinsic microporosity-1 (PIM-1). Purified TTSBI (2.56 g), TFTPN (1.51 g), and dried K_2CO_3 (2.08 g) were added to a three-necked flask, stirring for 0.5 h in a N_2 atmosphere. Anhydrous DMF (50 mL) was added to the reaction system. The reactant was heated to 65 ℃ until the monomers were dissolved entirely and kept for 72 h. After the reaction, deionized water was used to quench the reaction and precipitate the PIM-1 polymer. The crude product was collected by filtration and washed with additional deionized water. The PIM-1 crude product was purified by dissolving in chloroform and recrystallizing with methanol three times. Finally, the fluorescent yellow PIM-1 polymer was dried under vacuum at 80 °C for 12 h. The yield was ~75%.

1.10. Preparation of COF/PIM-1 and COF/polyimide (PI) MMMs. COF-based MMMs were prepared by incorporating TUS-621 or TUS-622 into the polymer matrix via a solution-casting method. Briefly, a predetermined amount of TUS-621 or TUS-622 was dispersed in 3 mL of chloroform, sonicated, and stirred for 8 h. Then, the polymer was added to the COF dispersion in portions (1:2:7) and stirred overnight. The resulting casting solution was poured into a flat-bottomed glass Petri dish and allowed to undergo slow solvent evaporation at ambient temperature to form a free-standing membrane. The obtained membranes were carefully peeled from the substrate and subsequently dried in a vacuum oven at 60 °C overnight to remove residual solvent.

1.11. Preparation of thin-film nanocomposite (TFN) membranes of TUS-621/Pebax-10% and TUS-622/Pebax-10%. Before coating the selective layer of COF-based MMMs, 3 wt% PDMS was first coated as a groove layer to smooth the surface and prevent pore penetration of the Pebax solution.

PDMS and TEOS were dissolved in n-heptane and stirred for 2 h. DBTDL was added and stirred for 30 min, and then sonicated for 30 min. The mass ratio of each component was W_{PDMS} : $W_{n\text{-}heptane}$: W_{TEOS} : W_{DBTDL} = 30 : 70 : 2.5 : 0.5. The PDMS solution was coated onto the PAN substrate using a roller doctor blade coater (RK print coater instrument, UK) at room temperature with a speed of 60 cm/min. The PDMS layer was cured by heating at 80 °C after evaporating the solvent at room temperature. The 2 wt% casting solution of the COF-based MMMs was coated onto the PAN substrate using the roller doctor blade coater at room temperature with a speed of 60 cm/min. The selective layer was cured by heating at 80 °C after evaporating the solvent at room temperature.

1.12. Calculation of Isosteric heat of adsorption (Q_{st})

A Virial equation comprising the temperature-independent parameters a_i and b_j was employed to calculate the enthalpies of adsorption for CO_2, CH_4, and H_2 in TUS-621 and TUS-622, which were measured at 273 and 298 K.

$$\ln P = \ln N + \frac{1}{T}\sum_{i}^{m} a_i N_i + \sum_{j}^{n} b_j N_j \tag{S1}$$

$$Qst = -R\sum_{i=0}^{m} a_i N_i \tag{S2}$$

Here, P is the pressure expressed in mmHg, N is the amount absorbed in mmol/g, T is the temperature in K, a_i and b_j are virial coefficients, and m, n represent the number of coefficients required to adequately describe the isotherms (herein, m =5 and n = 2). Q_{st} is the coverage-dependent isosteric heat of adsorption, and R is the universal gas constant.

1.13. Calculation of selectivity via ideal adsorption solution theory (IAST). The CO_2, CH_4, and H_2 adsorption isotherms were first fitted to a dual-site Langmuir–Freundlich (DSLF) model (S3):

$$q = \frac{q_{sat,A} b_A p^{\alpha_A}}{1 + b_A p^{\alpha_A}} + \frac{q_{sat,B} b_B p^{\alpha_B}}{1 + b_B p^{\alpha_B}} \tag{S3}$$

where q is the amount of adsorbed gas (mmol·g^{-1}), p is the bulk gas-phase pressure (bar), q_{sat} is the saturation amount (mmol·g^{-1}), b is the Langmuir–Freundlich parameter (bar$^{-\alpha}$), and α is the Langmuir–Freundlich exponent (dimensionless) for two adsorption sites A and B indicating the presence of weak and strong adsorption sites.

IAST is based on a Raoult's law–type relationship between the fluid and adsorbed phase:

$$P_i = P y_i = P_i^0 x_i \tag{S4}$$

$$\sum_{i=1}^{n} x_i = \sum_{i=1}^{n} \frac{P_i}{P_i^0} = 1 \tag{S5}$$

where P_i is the partial pressure of component i (bar), P is the total pressure (bar), and y_i and x_i represent the mole fractions of component i in the gas phase and adsorbed phase, respectively. P_i^0 is the equilibrium vapor pressure (bar).

In IAST, P_i^0 is defined by relating to spreading pressure (π) as:

$$\frac{\pi S}{RT} = \int_0^{P_i^0} \frac{q_i(P_i)}{P_i} dP_i = \pi \text{ (constant)} \tag{S6}$$

where S is the specific surface area of the adsorbent (m^2·g^{-1}), R is the gas constant (8.314 J·K^{-1}·mol^{-1}), T is the temperature (K), and $q_i\,(P_i)$ is the single component equilibrium obtained from isotherms (mmol·g^{-1}).

For a DSLF model, the integral in eq S6 has an analytical solution:

$$\int_0^{P_i^0} \frac{q_i(P_i)}{P_i} dP_i = \pi \text{ (constant)} = \frac{q_{sat,A}}{\alpha_A} \ln[1 + b_A(P_i^0)^{\alpha_A}] + \frac{q_{sat,B}}{\alpha_B} \ln[1 + b_B(P_i^0)^{\alpha_B}] \tag{S7}$$

The isotherm parameters are derived from the previous fitting. For a binary component system, the unknowns will be π, P_1^0, and P_2^0, which can be obtained by simultaneously solving equations S5 and S7.

The adsorbed amount of each compound in the mixture was calculated as:

$$q_i^{mix} = x_i q_t \tag{S8}$$

$$\frac{1}{q_T} = \sum_{i=1}^{n} \frac{x_i}{q_i(P_i^0)} \tag{S9}$$

where q_i^{mix} is the adsorbed amount of component *i* (mmol·g^{-1}), and q_t is the total adsorbed amount (mmol·g^{-1}).

The adsorption selectivities (S_{ads}) were calculated according to:

$$S_{ads} = \frac{q_1/q_2}{p_1/p_2} \tag{S10}$$

In this study, All IAST calculations were performed assuming an equimolar (50:50) binary gas mixture at 298 K and total pressures up to 1 atm.

1.14. Evaluation of gas permeation performance of MMMs. Gas permeation measurements of the MMMs were conducted using the Wicke–Kallenbach permeation technique to evaluate both single-gas (CO_2, CH_4, and H_2) and mixed-gas separation performance. Binary gas mixtures of CO_2/CH_4 (50/50, v/v) and CO_2/H_2 (50/50, v/v) were employed for mixed-gas permeation experiments. All permeation tests were carried out over a temperature range of 25–100 °C. During the measurements, the feed-side pressure was controlled between 2 and 10 bar, while the permeate side was maintained at atmospheric pressure. Argon was used as both the purge gas and carrier gas. The permeating gases were continuously transported to a gas chromatograph (GC) for quantitative analysis of gas composition. Calibration curves were established using 6–8 concentration points, selected based on the statistical analysis of more than 20 parallel measurements to ensure accuracy and reproducibility.

Gas permeability and selectivity were used as the primary parameters to assess membrane separation performance.

The gas permeability was calculated using Equation (S11):

$$P = \frac{N \times l}{\Delta P \times A} \tag{S11}$$

where P is the gas permeability (Barrer; 1 Barrer = 3.35 × 10^{-16} mol m^{-1} s^{-1} Pa^{-1}), N is the steady-state permeate molar flow rate (mol s^{-1}), l is the thickness of the membrane (μm), ΔP is the transmembrane pressure difference between the upstream and downstream sides (Pa), and A is the effective membrane area (m^2).

The ideal and mixed-gas selectivity ($\alpha_{i,j}$) was calculated according to Equation (S12):

$$\alpha_{i,j} = \frac{P_i}{P_j} \tag{S12}$$

where P_i and P_j are the permeabilities of gas components *i* and *j*, respectively.

All reported permeation and selectivity values represent the average of measurements obtained from at least three independently prepared membrane samples.

1.15. Diffusivity and solubility coefficients of Pebax and MMMs. Gas transport through the membranes was analyzed using the solution–diffusion model. The solubility coefficient, S (mol m^{-3} Pa^{-1}), was determined from the experimentally measured gas uptake. The gas uptake values, initially obtained in mmol g^{-1}, were converted to a volumetric basis (mmol cm^{-3}) using the membrane density. The solubility coefficient was then calculated according to Equation (S13):

$$S = \frac{C * \rho_m}{p} \tag{S13}$$

where C (mol·g^{-1}) is the gas uptake, ρ_m (g·m^{-3}) is the membrane density, and p (Pa) is the equilibrium pressure.

The diffusivity coefficient, D (m^2 s^{-1}), was calculated according to Equation (S14):

$$D = \frac{P}{S} \tag{S14}$$

where P is the gas permeability, expressed in Barrer (1 Barrer = 3.35 × 10^{-16} mol m^{-1} s^{-1} Pa^{-1}).

1.16. Computational details. All density functional theory (DFT) calculations were carried out using the open-source Quantum ESPRESSO package.[4-6] The exchange-correlation interactions were treated within the generalized gradient approximation (GGA) using the Perdew-Burke-Ernzerhof (PBE) functional.[7] All structures were fully optimized to obtain their ground-state energies, employing electronic and ionic convergence thresholds of 7.35 × 10^{-7} Ry (~10^{-5} eV) and 1 × 10^{-4} Ry (~0.001 eV), respectively. A force convergence criterion of 7.78 × 10^{-4} Ry/Bohr (0.02 eV/Å) was used for all geometry optimizations. Brillouin-zone sampling was performed using a 3 x 3 x 1 Monkhorst–Pack k-

point grid.[8] Gaussian smearing with a width of 0.01 Ry was applied to aid electronic convergence. Long-range dispersion interactions were incorporated using the DFT-D3 van der Waals correction with damping.[9,10] To prevent spurious interactions between periodic images, a vacuum spacing of approximately 20 Å was introduced along the z-direction.[11]

The Gibbs free energy change (ΔG) for calculating adsorption energies of the gases was calculated using:

$$\Delta G = \Delta E + \Delta ZPE - T\Delta S \quad (S15)$$

where ΔE is the DFT-calculated energy difference between the adsorbed intermediate and the pristine surface, ΔZPE represents the zero-point energy correction, and ΔS denotes the entropy change of the gas-phase and adsorbed species evaluated at 298.15 K.

2. Nitrogen sorption

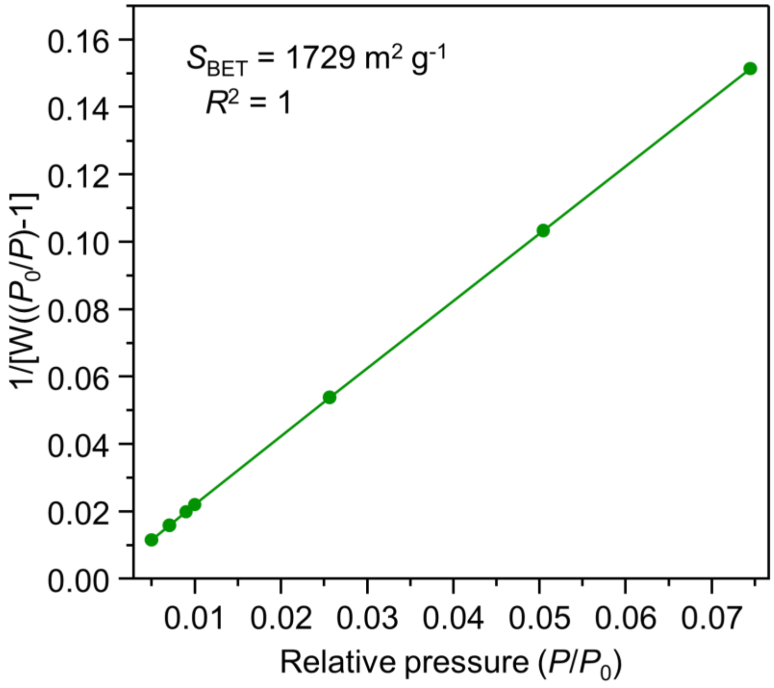

Figure S1. BET plot constructed from N_2 adsorption isotherms recorded at 77 K for TUS-621, yielding a BET surface area (S_{BET}) of 1729 m^2 g^{-1}.

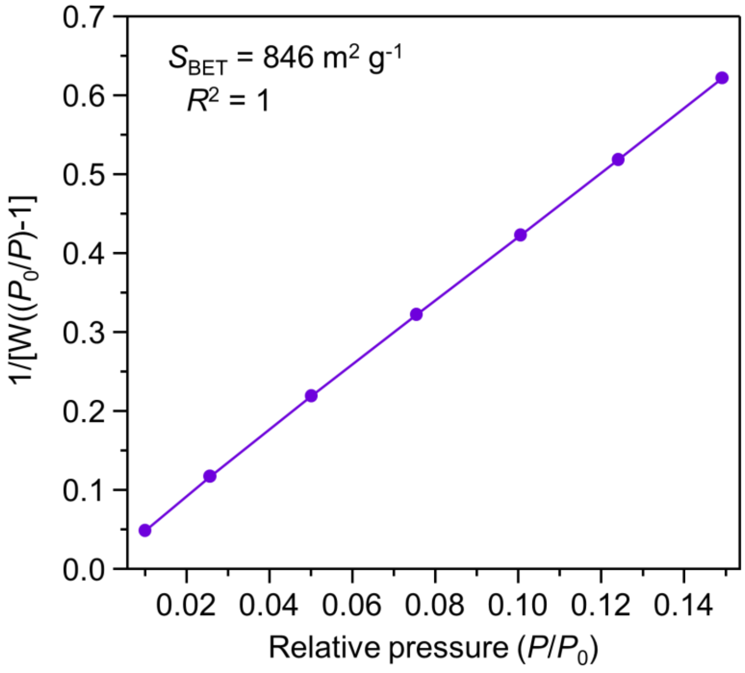

Figure S2. BET plot constructed from N_2 adsorption isotherms recorded at 77 K for TUS-622, yielding a BET surface area (S_{BET}) of 846 m^2 g^{-1}.

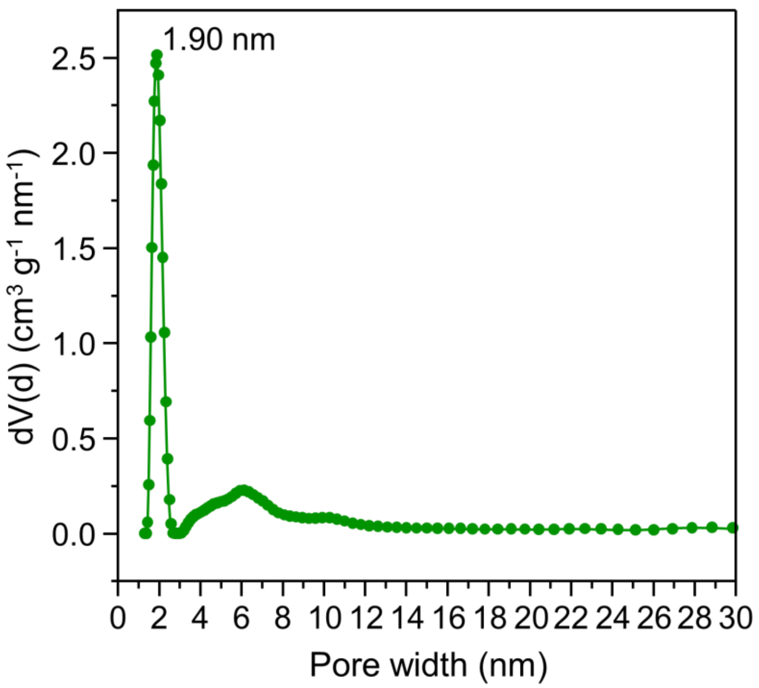

Figure S3. Pore size distribution profile of TUS-621.

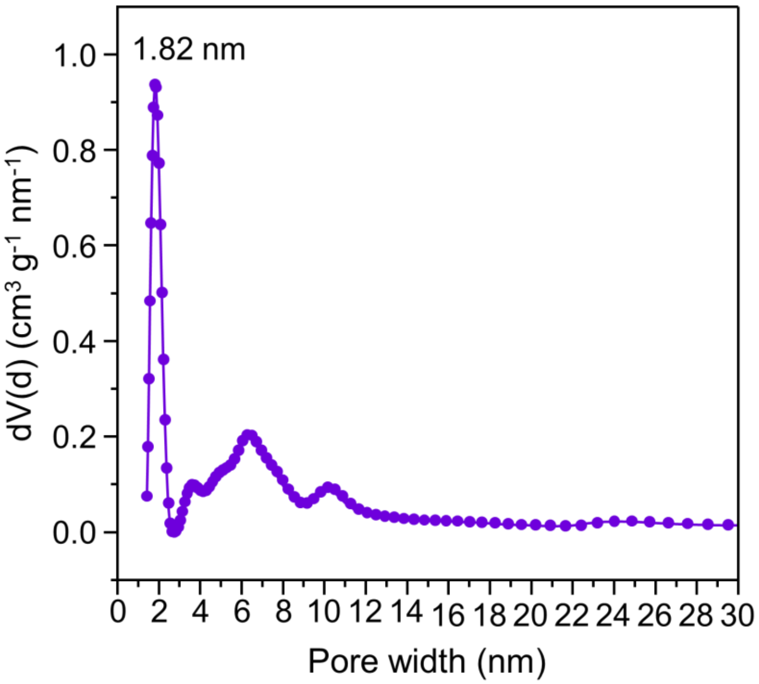

Figure S4. Pore size distribution profile of TUS-622.

The specific surface areas of TUS-621 and TUS-622 were evaluated from N_2 adsorption–desorption isotherms measured at 77 K using the BET Surface Identification (BETSI) software developed by the Adsorption and Advanced Materials Laboratory (AAML), Department of Chemical Engineering and Biotechnology, University of Cambridge. BETSI employs an automated, statistically grounded approach to objectively identify the appropriate pressure range for BET fitting, thereby reducing subjectivity commonly associated with manual analysis. All BET calculations were performed in strict accordance with the standardized BET evaluation protocol reported by Fairen-Jimenez et al. (2022),[12] which provides a rigorous and reproducible framework for surface-area determination in porous materials. This methodology is grounded in the Rouquerol consistency criteria, requiring that: (i) the BET plot exhibits linear behavior within the selected relative pressure range, (ii) the BET C constant remains positive, and (iii) monolayer adsorption is achieved within the chosen fitting window. Compliance with these criteria is widely recognized as essential for obtaining physically meaningful BET surface areas for COFs, MOFs, and other micro- and mesoporous adsorbents. The BETSI algorithm automatically reports the optimal fitting region together with associated statistical descriptors, calculated surface-area values, and corresponding uncertainty estimates. This data-driven approach ensures high reproducibility, minimizes user-induced bias, and enables transparent comparison across different porous systems. The complete set of fitting parameters and statistical outputs is provided below.

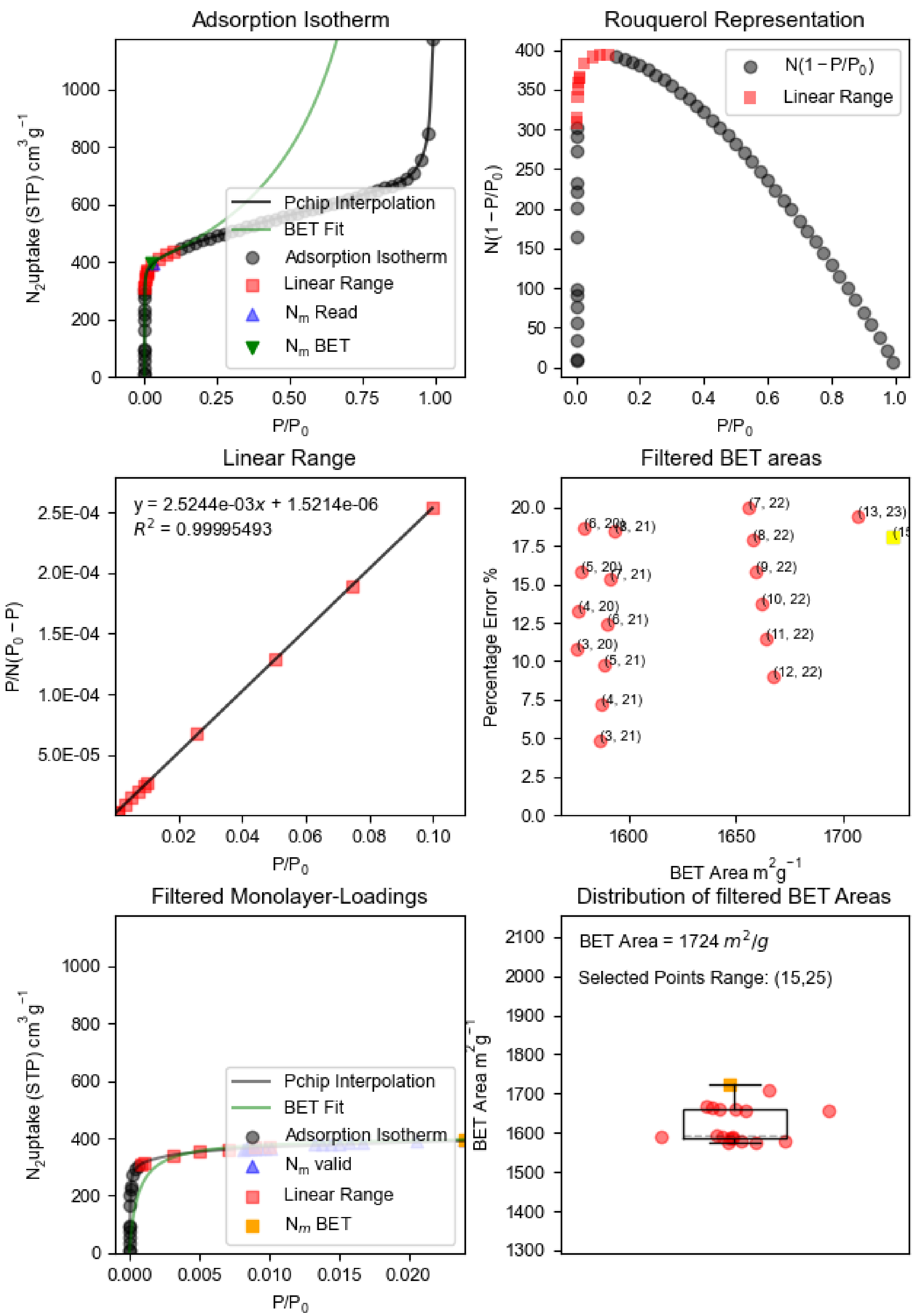
Adsorption Isotherm
N2uptake (STP) cm3 g−1
P/P0
Pchip Interpolation
BET Fit
Adsorption Isotherm
Linear Range
Nm Read
Nm BET
Rouquerol Representation
N(1−P/P0)
Linear Range
Linear Range
y = 2.5244e-03x + 1.5214e-06
R2 = 0.99995493
P/N(P0−P)
Filtered BET areas
Percentage Error %
BET Area m2g−1
Filtered Monolayer-Loadings
Nm valid
Distribution of filtered BET Areas
BET Area = 1724 m2/g
Selected Points Range: (15,25)
BET Area m2g−1

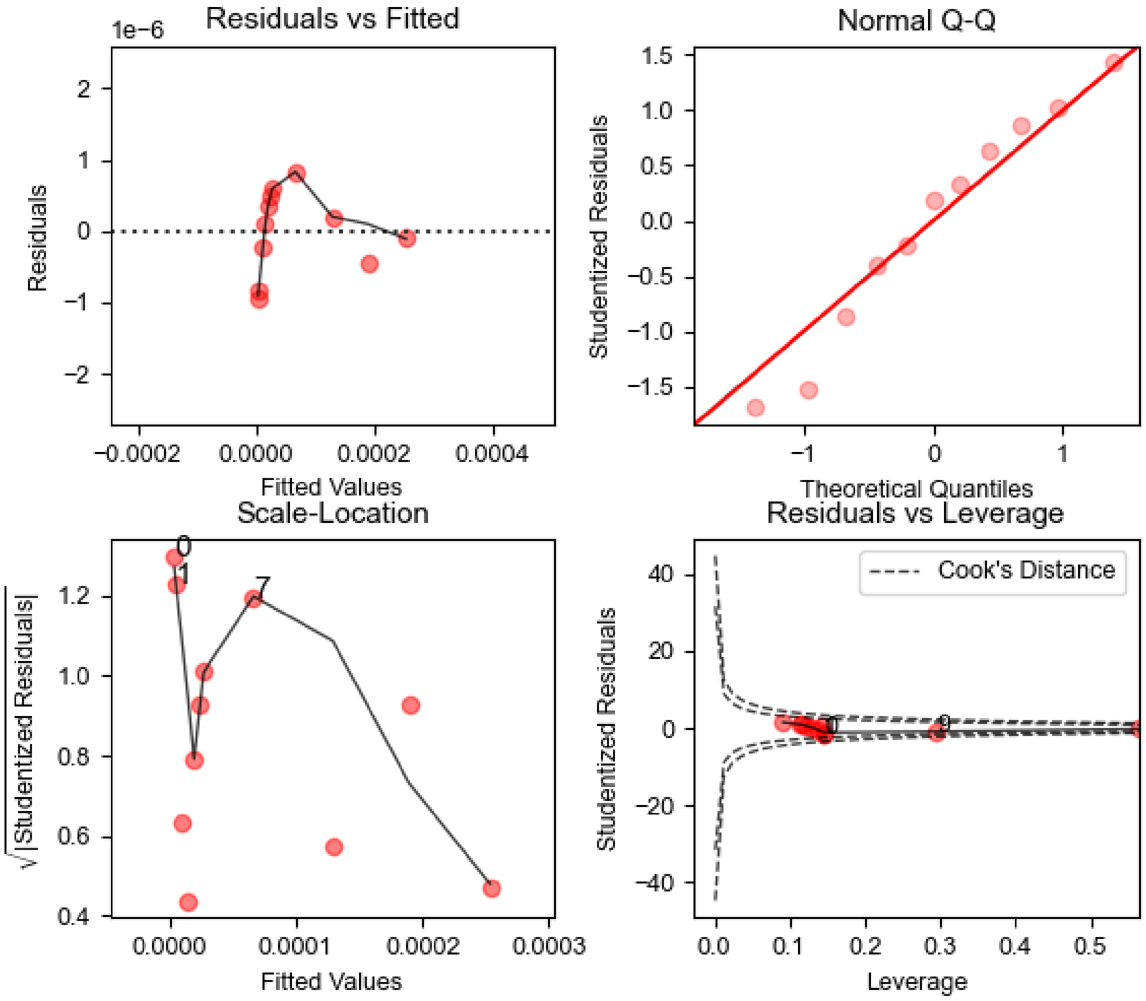

Figure S5. BETSI analysis illustrating the selected BET fitting region and associated fitting parameters for TUS-621.

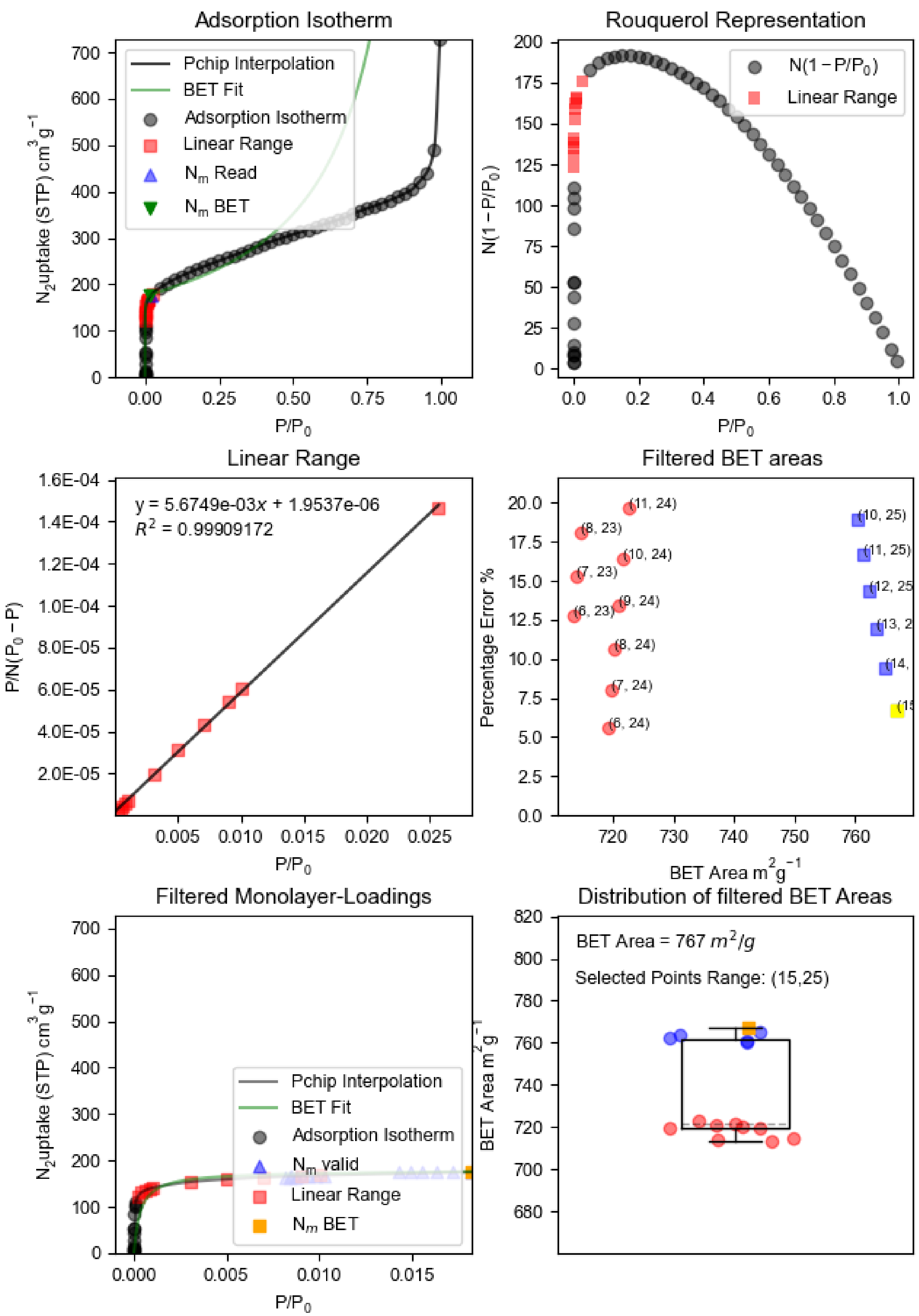
Adsorption Isotherm
Pchip Interpolation
BET Fit
Adsorption Isotherm
Linear Range
N_m Read
N_m BET
N_2uptake (STP) $cm^3 g^{-1}$
P/P_0
Rouquerol Representation
$N(1-P/P_0)$
Linear Range
Linear Range
$y = 5.6749e\text{-}03x + 1.9537e\text{-}06$
$R^2 = 0.99909172$
$P/N(P_0-P)$
Filtered BET areas
(11, 24)
(8, 23)
(10, 24)
(7, 23)
(9, 24)
(6, 23)
(8, 24)
(7, 24)
(6, 24)
(10, 25)
(11, 25)
Percentage Error %
BET Area $m^2 g^{-1}$
Filtered Monolayer-Loadings
N_m valid
Distribution of filtered BET Areas
BET Area = 767 m^2/g
Selected Points Range: (15,25)

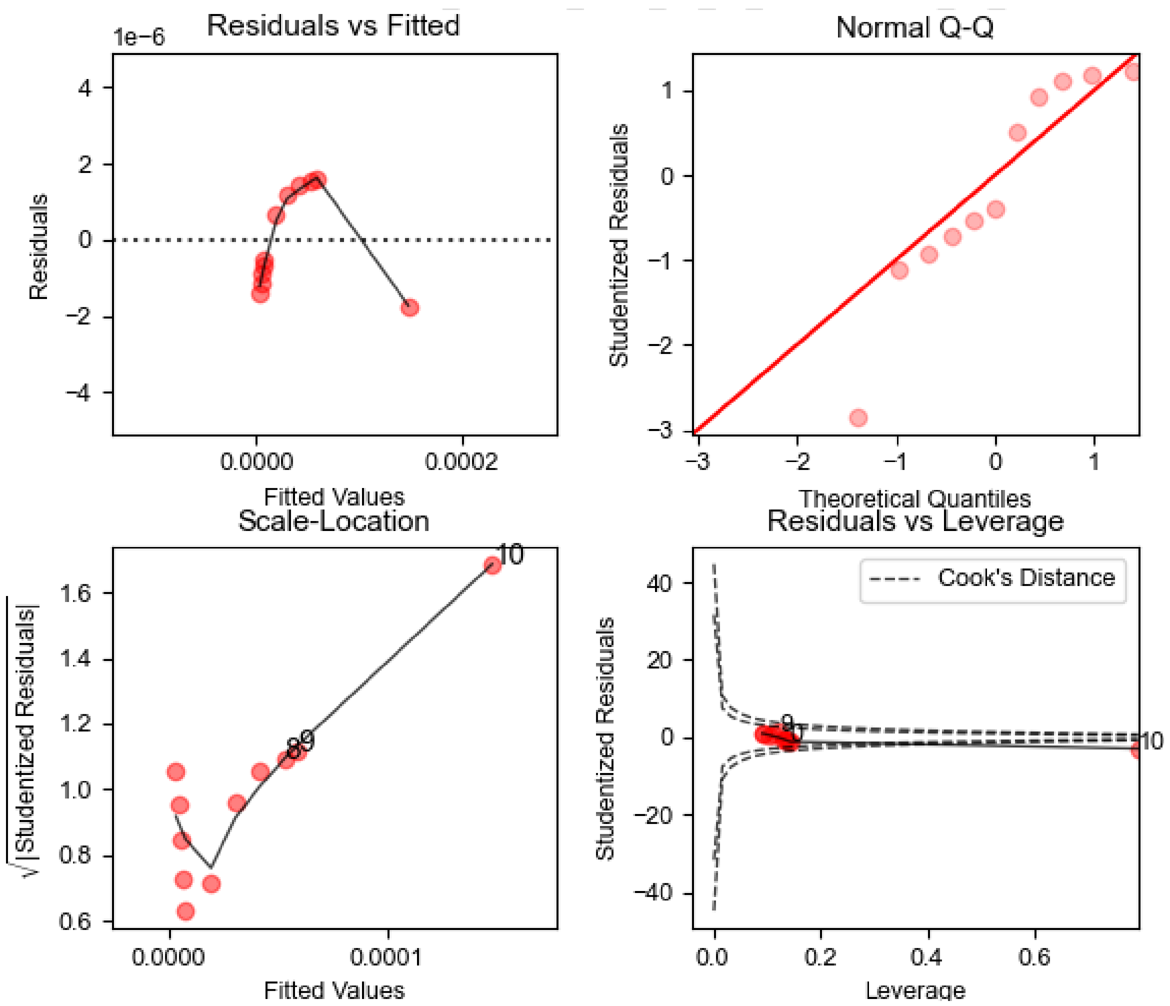

Figure S6. BETSI analysis illustrating the selected BET fitting region and associated fitting parameters for TUS-622.

3. Scanning electron microscopy (SEM)

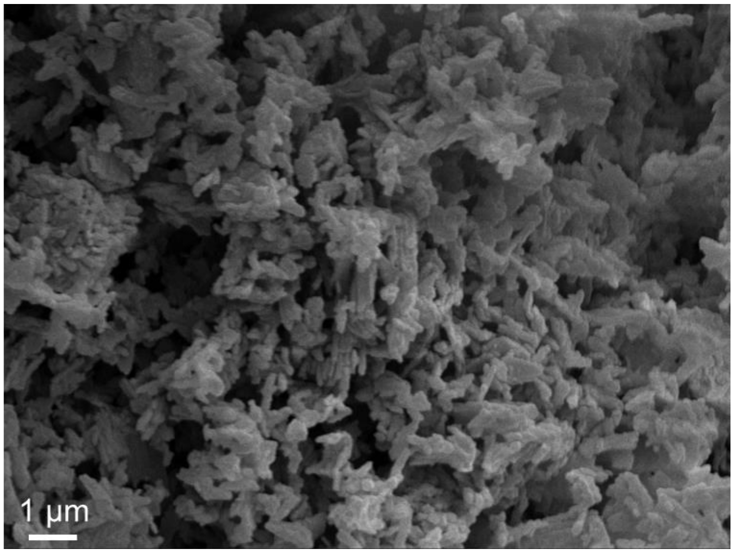

Figure S7. SEM micrograph of TUS-621.

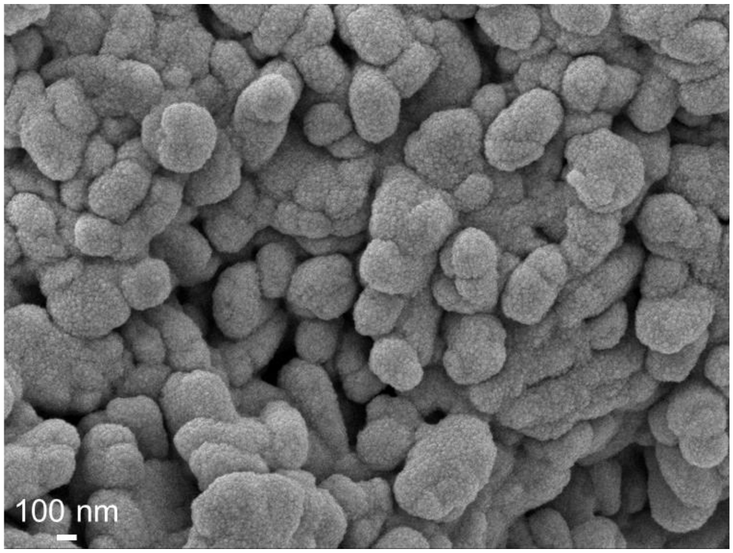

Figure S8. SEM micrograph of TUS-622.

4. Transmission electron microscopy (TEM)

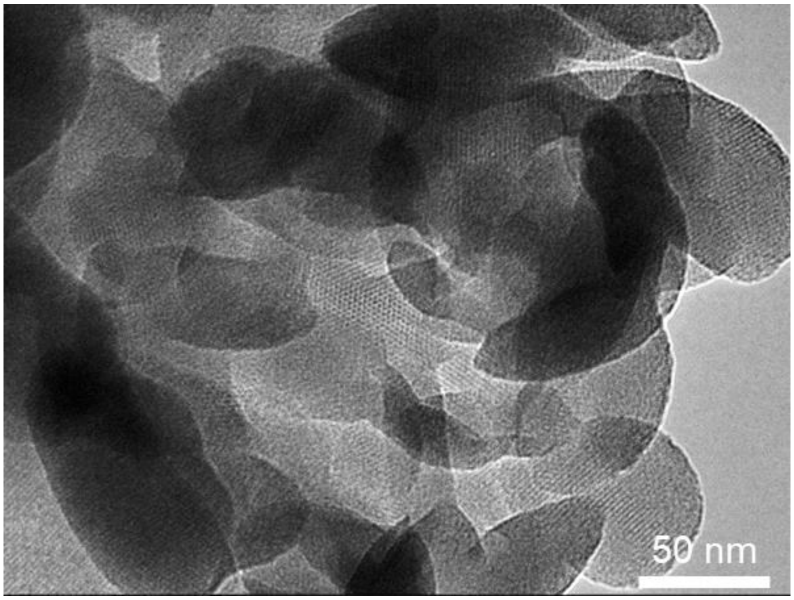

Figure S9. HR-TEM image of TUS-621.

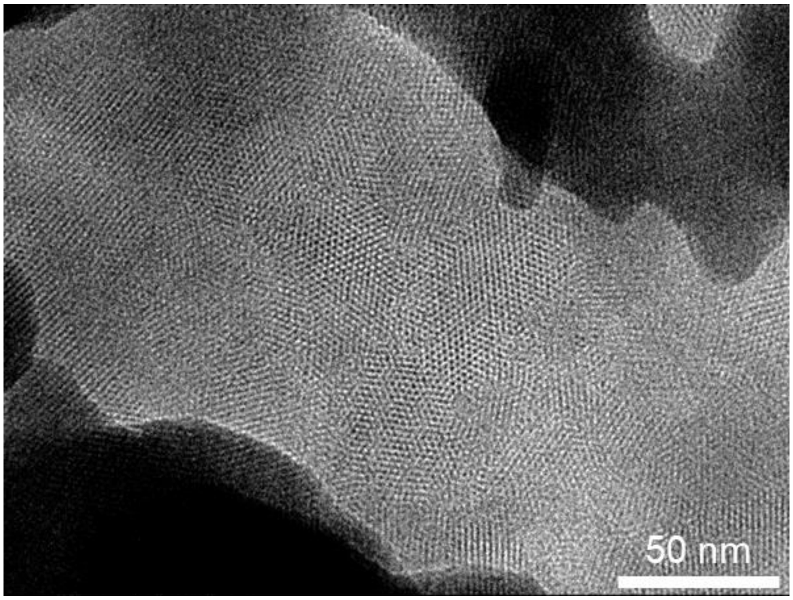

Figure S10. HR-TEM image of TUS-622.

5. Thermogravimetric analysis (TGA)

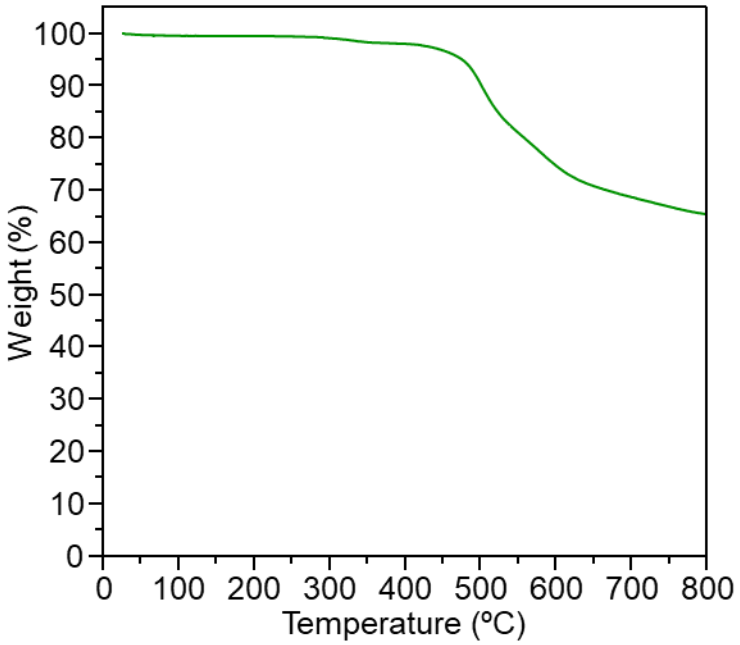

Figure S11. TGA curve of TUS-621 obtained under a nitrogen atmosphere.

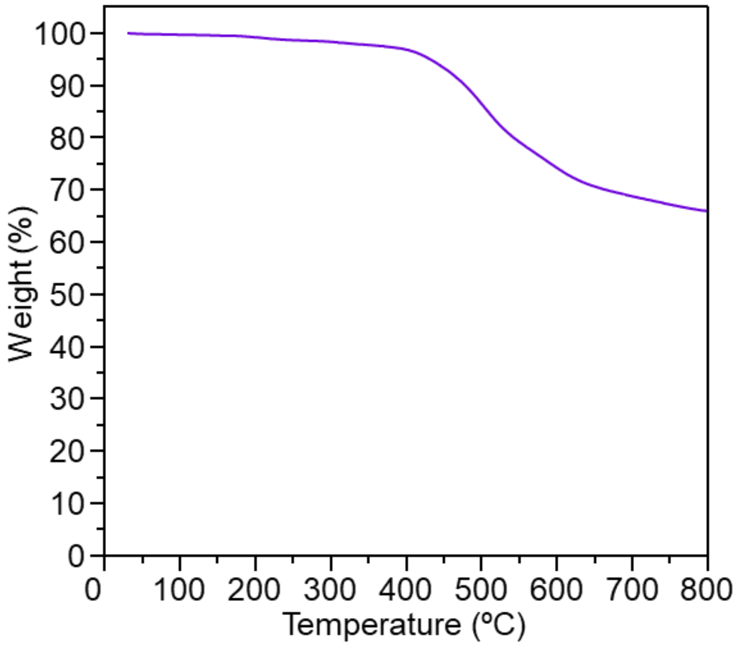

Figure S12. TGA curve of TUS-622 obtained under a nitrogen atmosphere.

6. Chemical stability analysis

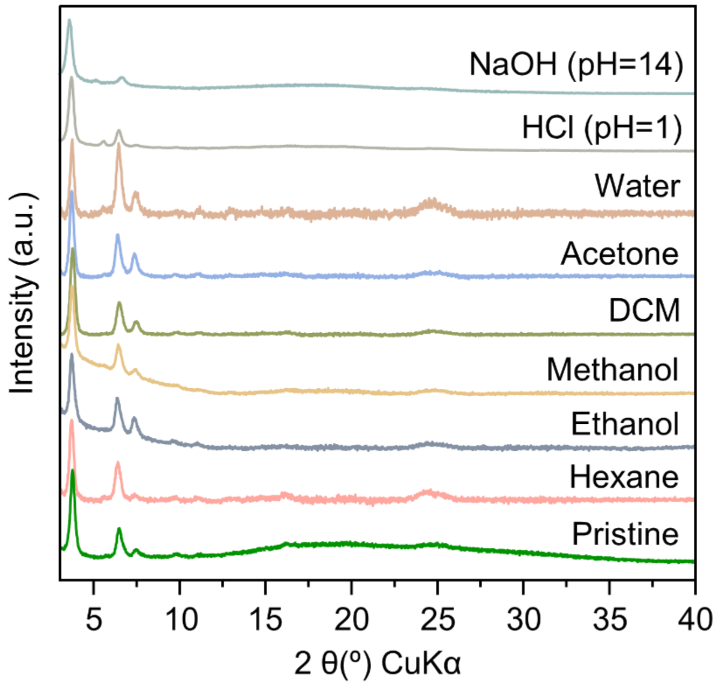

Figure S13. PXRD patterns of TUS-621 after 24 h immersion in various solvents, including acidic and alkaline media.

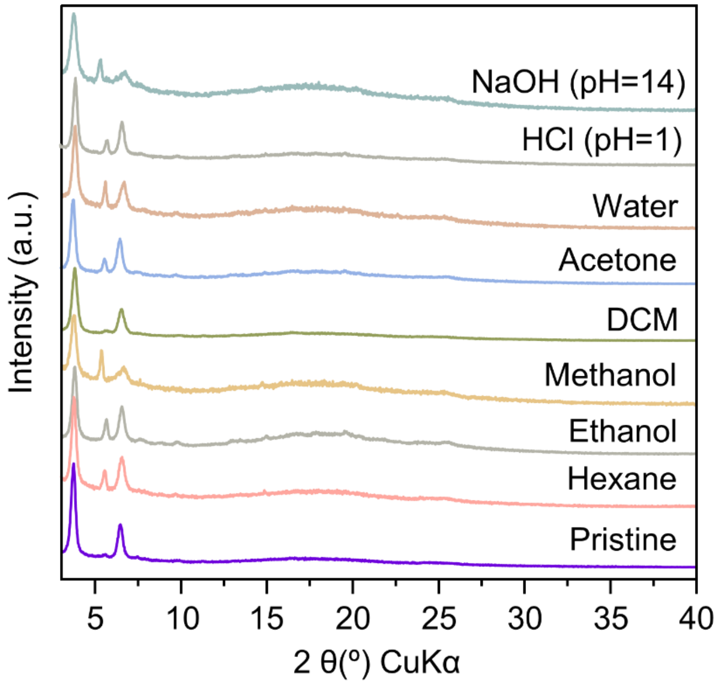

Figure S14. PXRD patterns of TUS-622 after 24 h immersion in various solvents, including acidic and alkaline media.

7. Q_{st} of TUS-621 and TUS-622

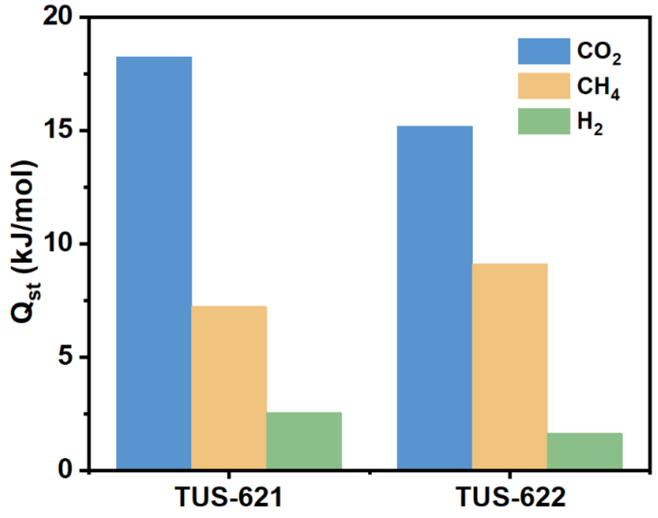

Figure S15. Q_{st} of TUS-621 and TUS-622 at near-zero coverage.

Table S1. Q_{st} of TUS-621 and TUS-622 at near-zero coverage.

Q_{st} (kJ/mol)	TUS-621	TUS-622
CO_2	18.23	15.18
CH_4	7.22	9.10
H_2	2.54	1.63

8. Ideal Adsorbed Solution Theory (IAST)–based selectivity calculations

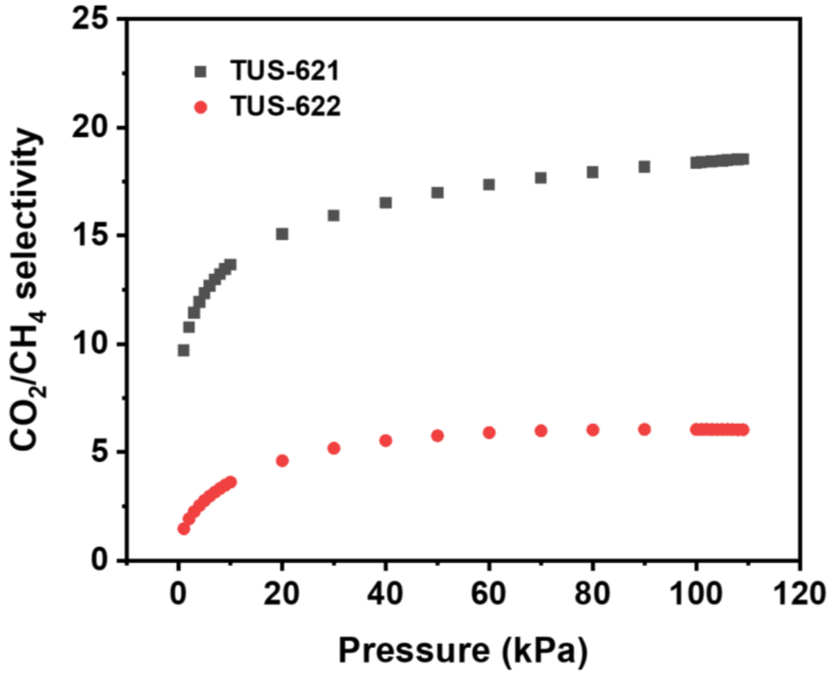

Figure S16. Pressure-dependent CO_2/CH_4 adsorption selectivity of TUS-621 and TUS-622 calculated using IAST based on single-component adsorption isotherms.

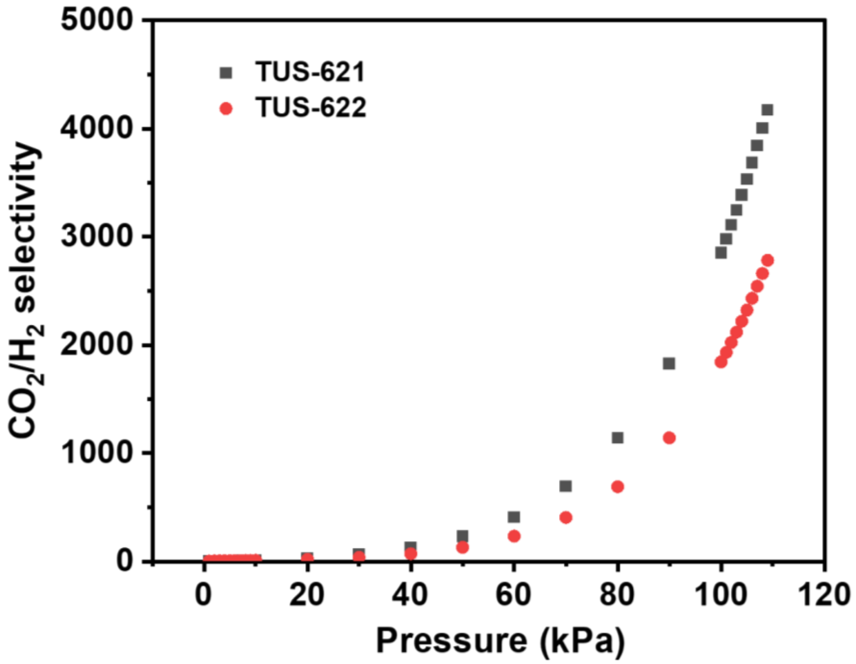

Figure S17. Pressure-dependent CO_2/H_2 adsorption selectivity of TUS-621 and TUS-622 calculated using IAST based on single-component adsorption isotherms.

9. Membrane thickness

Table S2. Thicknesses of pristine Pebax and TUS-621– and TUS-622–based MMMs with varying filler loadings used in this study.

Membrane	Thickness (μm)
Pebax	61 ± 3.5
TUS-621/Pebax-5%	53 ± 2.6
TUS-621/Pebax-10%	47 ± 1.7
TUS-621/Pebax-15%	51 ± 2.1
TUS-622/Pebax-5%	59 ± 3.3
TUS-622/Pebax-10%	52 ± 2.0
TUS-622/Pebax-15%	55 ± 2.8

10. SEM images and mapping (top-view and cross-section)

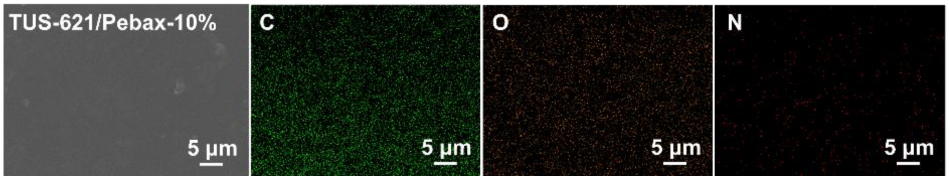

Figure S18. Top-view SEM image and corresponding EDS elemental mapping of C, O, and N for the TUS-621/Pebax-10% MMM.

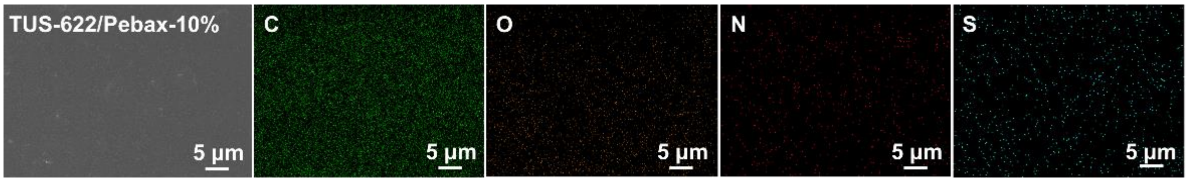

Figure S19. Top-view SEM image and corresponding EDS elemental mapping of C, O, N and S for the TUS-622/Pebax-10% MMM.

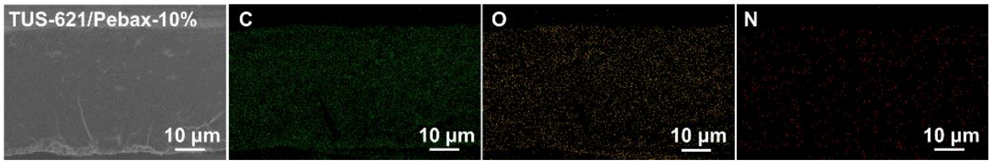

Figure S20. Cross-section SEM image and corresponding EDS elemental mapping of C, O, and N for the TUS-621/Pebax-10% MMM.

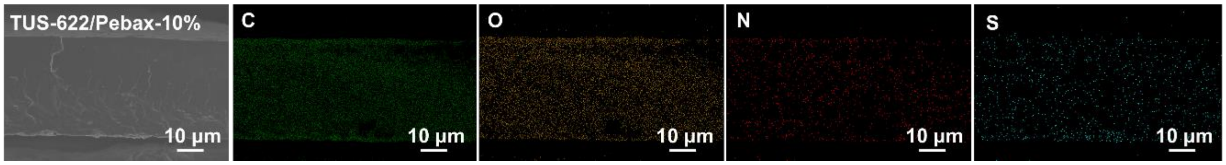

Figure S21. Cross-section SEM image and corresponding EDS elemental mapping of C, O, N and S for the TUS-622/Pebax-10% MMM.

11. TEM of TUS-621/Pebax-10% and TUS-622/Pebax-10%

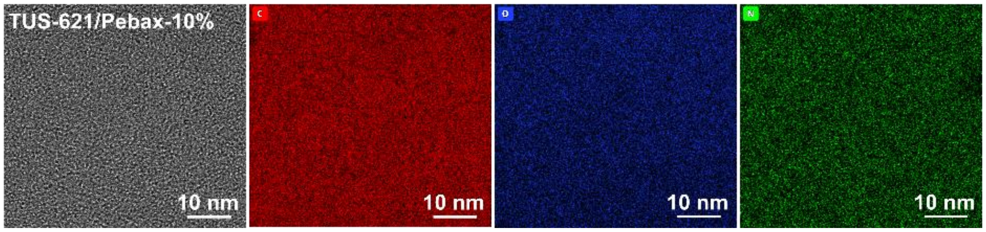

Figure S22. TEM image and corresponding EDS elemental mapping of C, O, and N for the TUS-621/Pebax-10% MMM.

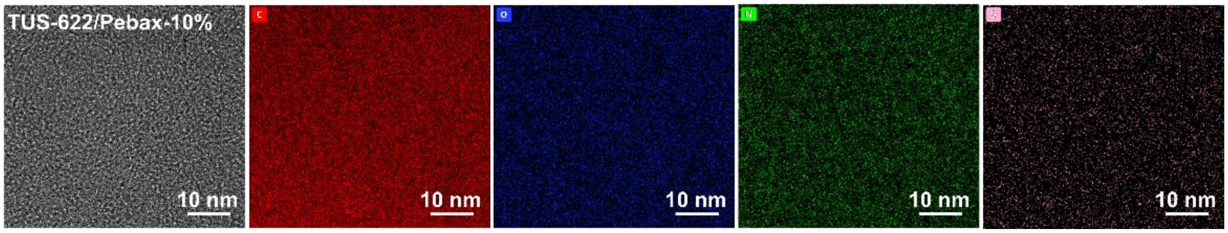

Figure S23. TEM image and corresponding EDS elemental mapping of C, O, N, and S for the TUS-622/Pebax-10% MMM.

12. XRD patterns of membranes

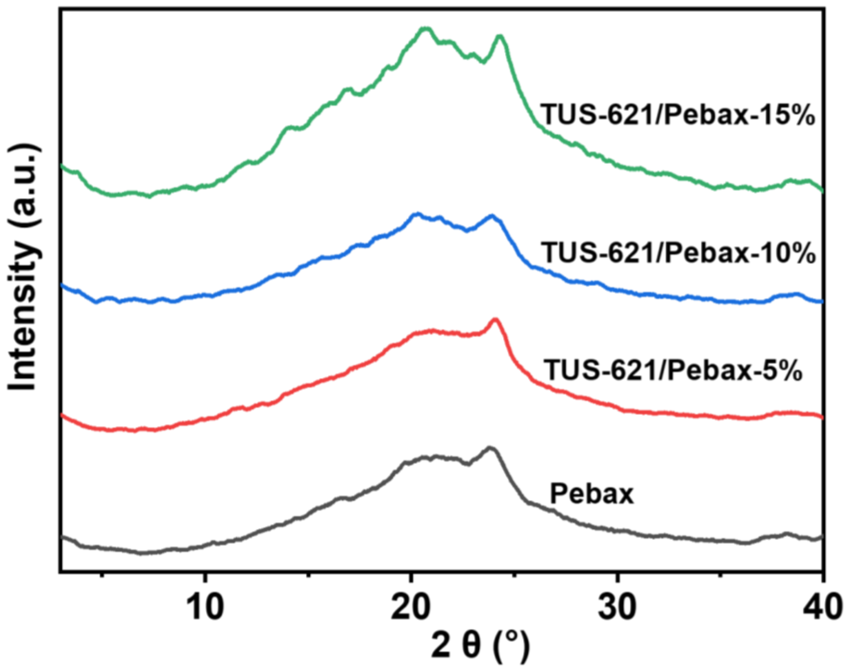

Figure S24. XRD patterns of pristine Pebax and TUS-621/Pebax MMMs at varying filler contents.

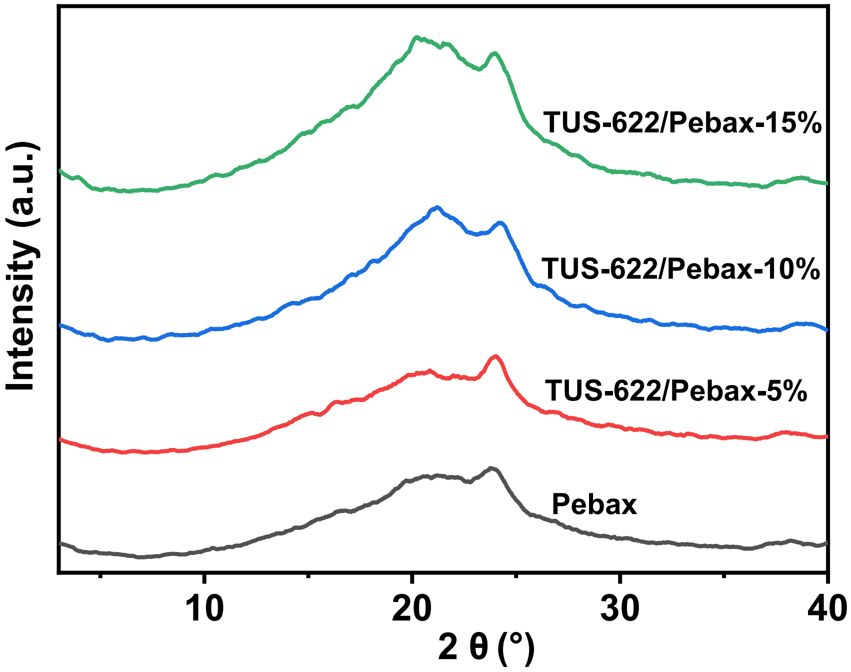

Figure S25. XRD patterns of pristine Pebax and TUS-622/Pebax MMMs at varying filler contents.

13. FT-IR spectra of membranes

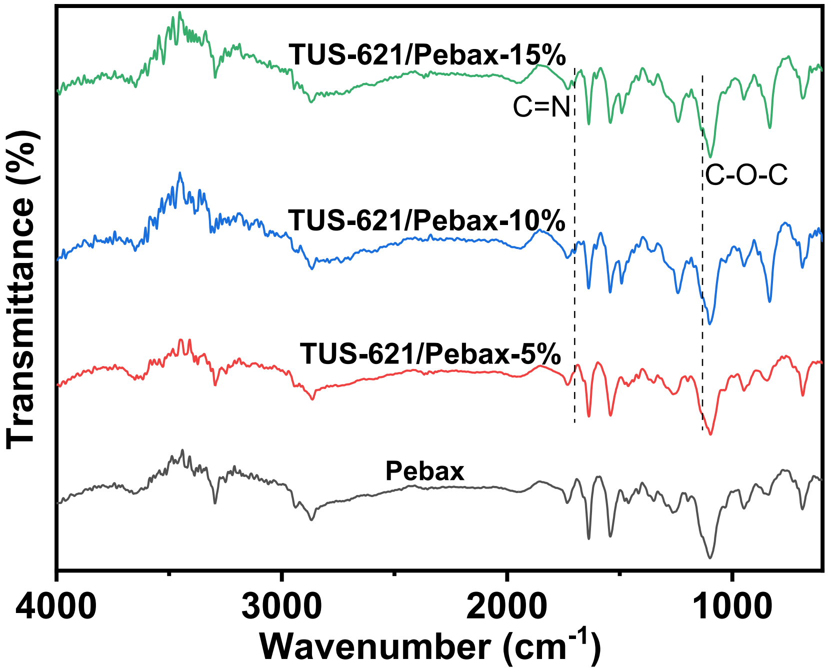

Figure S26. FT-IR spectra of pristine Pebax and TUS-621/Pebax MMMs at varying filler contents.

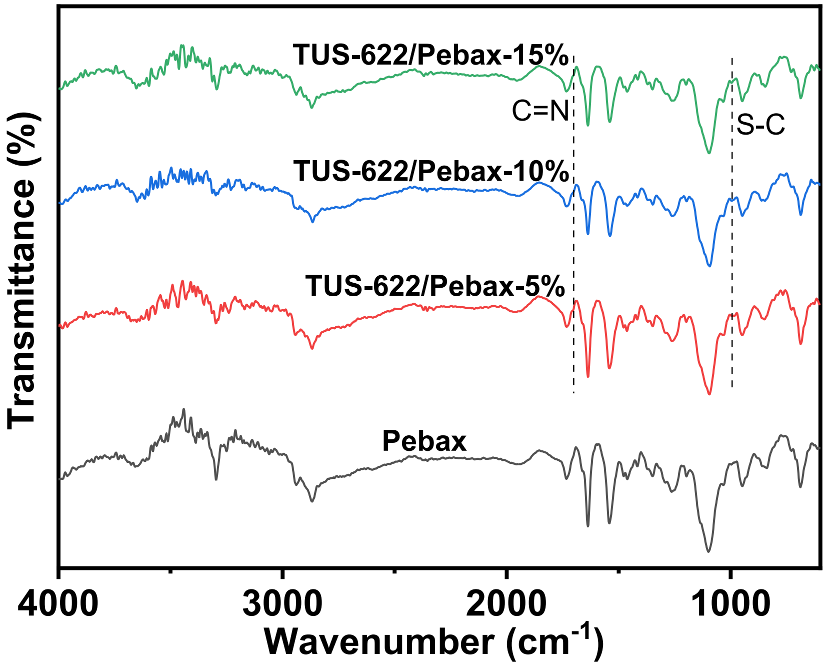

Figure S27. FT-IR spectra of pristine Pebax and TUS-622/Pebax MMMs at varying filler contents.

14. Tensile test of Pebax and MMMs

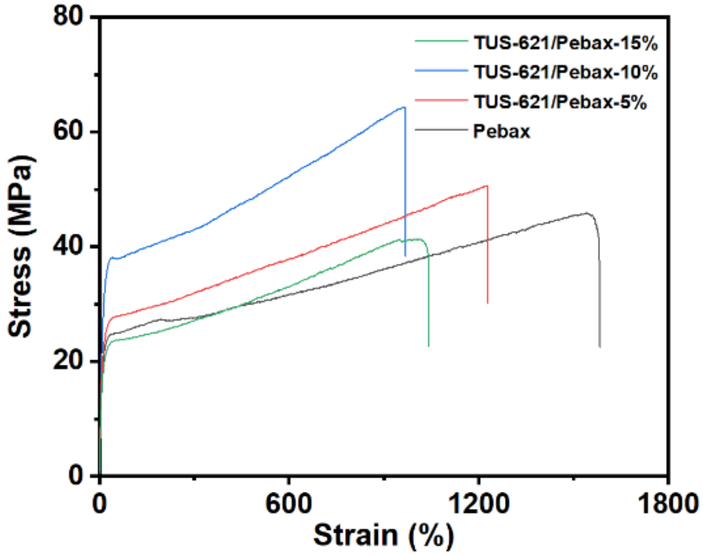

Figure S28. Tensile tests of Pebax and TUS-621–based MMMs.

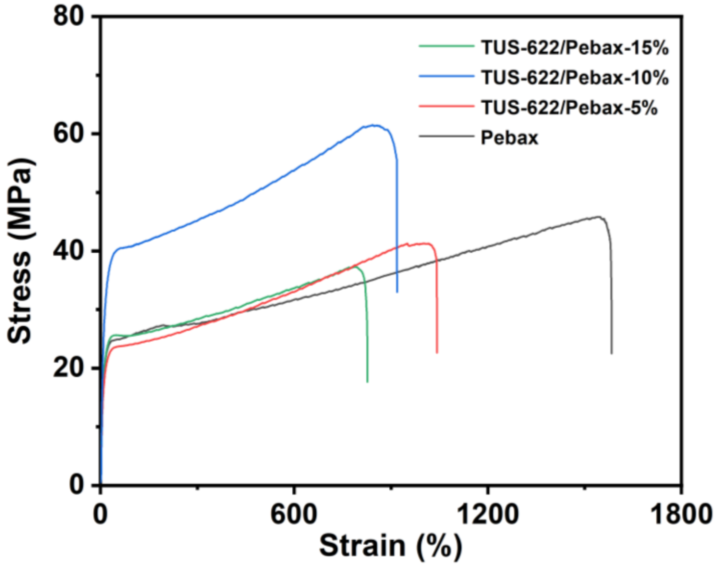

Figure S29. Tensile tests of Pebax and TUS-622–based MMMs.

Table S3. Young’s modulus of Pebax and TUS–based MMMs.

Membranes	Young's modulus (MPa)
Pebax	2.96
TUS-621/Pebax-5%	4.13
TUS-621/Pebax-10%	6.69
TUS-621/Pebax-15%	4.06
TUS-622/Pebax-5%	4.10
TUS-622/Pebax-10%	7.07
TUS-622/Pebax-15%	4.74

15. Single-gas permeation performance

Table S4. Single-gas permeation properties of pristine Pebax and TUS-621– and TUS-622–based MMMs measured at 2 bar and 25 ºC.

Membrane	CO_2 permeability (Barrer)‡	CH_4 permeability (Barrer)‡	H_2 permeability (Barrer)‡	CO_2/CH_4 selectivity	CO_2/H_2 selectivity
Pebax	125.0 ± 8.6	10.00 ± 0.39	13.70 ± 0.27	12.50	9.12
TUS-621/Pebax-5%	308.3 ± 5.6	15.77 ± 0.41	19.77 ± 0.49	19.54	15.59
TUS-621/Pebax-10%	428.9 ± 11.2	8.60 ± 0.07	17.25 ± 0.67	49.87	24.86
TUS-621/Pebax-15%	478.5 ± 27.0	15.67 ± 0.20	21.78 ± 0.73	30.53	21.96
TUS-622/Pebax-5%	172.0 ± 14.1	9.79 ± 0.16	13.61 ± 0.49	17.56	12.63
TUS-622/Pebax-10%	210.0 ± 40.2	7.96 ± 0.07	8.84 ± 0.08	26.38	23.75
TUS-622/Pebax-15%	243.0 ± 11.0	9.34 ± 0.31	24.40 ± 1.83	26.02	9.95

‡Permeability values were calculated by multiplying the measured gas permeance by the corresponding membrane thickness.

‡1 Barrer = 3.347×10^{-16} mol·m^{-1}·s^{-1}·Pa^{-1}

16. D and S of Pebax and MMMs

Table S5. Solubility and Diffusivity of Pebax, TUS-621/Pebax-10% and TUS-622/Pebax-10% membranes.

Membrane	Solubility[a]		$S_{CO2/CH4}$	Diffusivity[b]		$D_{CO2/CH4}$
	CO_2	CH_4		CO_2	CH_4	
Pebax	4.83	1.10	4.40	0.87	0.31	2.84
TUS-621/Pebax-10%	8.25	0.84	9.34	1.74	0.33	5.34
TUS-622/Pebax-10%	7.35	0.75	9.80	0.96	0.36	2.69

[a]10^{-6} mol m^{-3} Pa^{-1}; [b]10^{-8} cm^2 s^{-1}

Table S6. Solubility and Diffusivity of Pebax, TUS-621/Pebax-10% and TUS-622/Pebax-10% membranes.

Membrane	Solubility[a]		$S_{CO2/H2}$	Diffusivity[b]		$D_{CO2/H2}$
	CO_2	H_2		CO_2	H_2	
Pebax	4.83	0.11	45.08	0.87	4.3	0.20
TUS-621/Pebax-10%	8.25	0.05	162.2	1.74	11	0.15
TUS-622/Pebax-10%	7.35	0.06	130.6	0.96	5.3	0.18

[a]10^{-6} mol m^{-3} Pa^{-1}; [b]10^{-8} cm^2 s^{-1}

17. Comparison of gas separation performance with previously reported membranes

Table S7. Benchmark comparison of CO_2/CH_4 selectivity and CO_2 permeability for TUS-621– and TUS-622–based MMMs reported in this work and in representative literature under comparable test conditions.

Membrane	Filler loading	Test condition	Thickness (μm)	CO_2 permeability (Barrer)	CO_2/CH_4 selectivity	Ref
Matrimid		35 °C, 4 bar	32	6.8	30.5	13
ACOF-1/Matrimid	16 wt %	35 °C, 4 bar	47	15.3	32.4	13
MOF/COF-16/Pebax	3 wt%	25 °C, 2 bar	124	815.9	20.3	14
TPDH-COF/Pebax	3 wt%	25 °C, 2 bar	96	813.6	28.3	15
MOF@COF/Psf	5 wt%	25 °C, 1 bar	53	7.1	46.7	16
NUS-2 COF/Ultem	20 wt%	25 °C, 2 bar	73	4.9	22.4	17
NUS-3 COF/Ultem	20 wt%	25 °C, 2 bar	100	15	28.3	17
Dha Tab-COF/Pebax	2 wt%	25 °C, 2 bar	1	295.8	21.6	18
NH_2-ZIF-8/6FDA-DAM	30 wt%	35 °C, 2 bar	58	1225.9	35.1	19
UiO-66-mod/PEI	10 wt%	25 °C, 1 bar	12.9	436.7	33	20
Pebax		25 °C, 2 bar	21	81	17.8	21
ZIF-L(Co)@ZIF-8/Pebax	3 wt%	25 °C, 2 bar	24	145.7	40.9	21
Pebax		25 °C, 2 bar	35.5	97.3	15.5	22
ns-MFI/Pebax	5 wt%	25 °C, 2 bar	39.7	159.1	27.4	22
MUF-15-F/6FDA-DAM	30 wt%		84	1300	37.1	23
Pebax				95.4	12.2	24
F-Ce/Pebax	8 wt%			1508	37	24
Pebax		25 °C, 2 bar	61	103	15.1	This work
TUS-621/Pebax	10 wt%	25 °C, 2 bar	47	433	55.3	This work
TUS-622/Pebax	10 wt%	25 °C, 2 bar	52	256	35.1	This work

Table S8. Benchmark comparison of CO_2/H_2 selectivity and CO_2 permeability for TUS-621– and TUS-622–based MMMs reported in this work and in representative literature under comparable test conditions.

Membrane	Filler loading	Test condition	Thickness (μm)	CO_2 permeability (Barrer)	CO_2/H_2 selectivity	Ref
COF_L/PVAm	10 wt%	25 ºC, 1.5 bar	0.145	221.1	16.1	25
MIL-53/Poly(ionic liquid)	2 wt%	30 ºC, 1.7 bar	180	92.7	13.3	26
Pebax		35 ºC, 4 bar	96	87.6	9.6	27
GO/ZIF-8@ZIF-67/Pebax	5 wt%	35 ºC, 4 bar	100	173.2	11.6	27
Pebax		25 ºC, 4 bar	68	92.4	9.3	28
GO/IL/Pebax	0.2 wt%	25 ºC, 4 bar	72	143	13.8	28
GO/IL/Pebax	0.5 wt%	25 ºC, 4 bar	0.3	271.6	5.8	28
F127-Tpy-M/Pebax	60 wt%	35 ºC, 1 bar	105	442.18	10.03	29
GO/Cross-linked PEO	1 wt%	35 ºC, 3.5 bar	180	474	9.9	30
XLPEO/Zn/Co-ZIF	15 wt%	35 ºC, 4 bar	100	761.9	8.4	31
PDMS/GO	0.3 wt%	20 ºC, 2 bar	350	3670	11.7	32
Z32-IP/Pebax		30 ºC, 2 bar	11.3	261	10	33
PIL–IL		20 ºC, 1 bar	86	324.7	11.4	34
PVA-IL53	53 wt%	25 ºC, 2 bar	104.4	66.9	7.73	35
UiO-66-NH_2-PVP-PEI	18 wt%	25 ºC, 1 bar	65	393.7	12.7	36
PIL–60IL $C(CN)_3$	60 wt%	35 ºC, 1 bar	105	505	12.2	37
Pebax/TCP	80 wt%	35 ºC, 1 bar		1673.2	6.4	38
PEBA/MOF-199@SBA-15	5 wt%	25 ºC, 1 bar		61.08	15.73	39
SIPN@GeFSIX-1-Cu	3 wt%	25 ºC, 3 bar	180	786	11.5	40
Pebax		25 ºC, 2 bar	61	97	9.3	This work
TUS-621/Pebax	10 wt%	25 ºC, 2 bar	47	407	25.2	This work
TUS-622/Pebax	10 wt%	25 ºC, 2 bar	52	178	23.2	This work

18. CO_2/CH_4 mixture gas separation performance of COF/PIM-1 and COF/PI MMMs

Table S9. CO_2 permeability and CO_2/CH_4 mixed-gas separation selectivity of pristine polymer and TUS-621– and TUS-622–based MMMs measured at 2 bar and 25 ºC.

Membranes	CO_2 permeability (Barrer)	CO_2/CH_4 selectivity
PIM-1	4654	18.1
TUS-621/PIM-1-10%	6735	32.3
TUS-622/PIM-1-10%	5436	25.6
PI	51.2	32.0
TUS-621/PI-10%	83.7	53.2
TUS-622/PI-10%	72.1	49.3

19. SEM characterization and mixed-gas separation performance of TUS-621/Pebax-10% and TUS-622/Pebax-10%TFN membranes

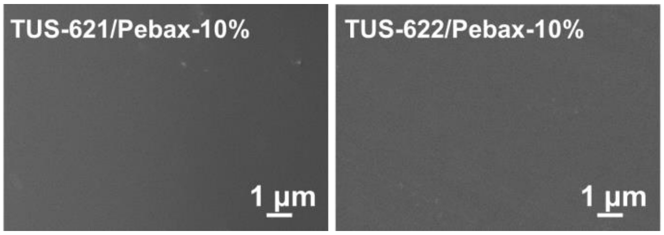

Figure S30. Top-view SEM images of TUS-621/Pebax-10% and TUS-622/Pebax-10% TFN membranes.

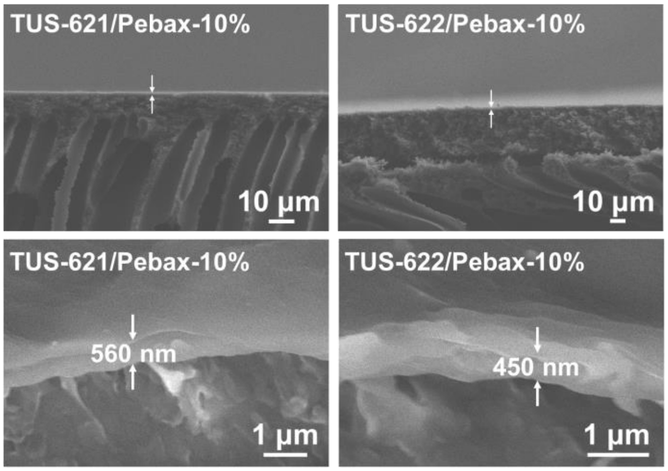

Figure S31. Cross-section SEM images of TUS-621/Pebax-10% and TUS-622/Pebax-10% TFN membranes.

Table S10. CO_2 permeance and CO_2/CH_4 mixed-gas separation selectivity of TUS-621/Pebax-10% and TUS-622/Pebax-10% TFN membranes measured at 2 bar and 25 ºC.

	**CO_2 permeance (GPU)**	**CO_2/CH_4 selectivity**
TFN (TUS-621/Pebax-10%)	932	33.5
TFN (TUS-622/Pebax-10%)	819	28.2

Table S11. CO_2 permeance and CO_2/H_2 mixed-gas separation selectivity of TUS-621/Pebax-10% and TUS-622/Pebax-10% TFN membranes measured at 2 bar and 25 ºC.

	**CO_2 permeance (GPU)**	**CO_2/H_2 selectivity**
TFN (TUS-621/Pebax-10%)	956	21.4
TFN (TUS-622/Pebax-10%)	797	18.5

20. Unit cell information and fractional atomic coordinates

Table S12. Unit cell information and fractional atomic coordinates of TUS-621.

Space group		*P*3	
Calculated unit cell		$a = b = 27.1616$ Å, $c = 4.8083$ Å, $\alpha = \beta = 90^{o}$, $\gamma = 120^{o}$	
Measured unit cell		$a = b = 27.1603$ Å, $c = 4.8127$ Å, $\alpha = \beta = 90^{o}$, $\gamma = 120^{o}$	
Pawley refinement		$R_p = 2.61\%$, $R_{wp} = 3.35\%$	
Atoms	x	y	z
C1	-1.28178	-0.61393	1.09258
C2	-1.23262	-0.56205	1.02242
C3	-1.23444	-0.51235	0.96208
C4	-1.28642	-0.51296	1.00348
C5	-0.33432	-0.615	1.10492
C6	-0.33375	-0.56285	1.09862
C7	-1.29756	-0.46784	0.90224
C8	-1.26544	-0.41104	0.98861
C9	-0.34064	-0.48255	2.70336
C10	-1.27549	-0.3696	0.87357
C11	-0.35051	-0.44133	2.58785
C12	-1.31768	-0.38434	0.67027
C13	-1.32682	-0.34011	0.54513
N14	-0.36509	-0.35331	2.35295
N15	-0.36178	-0.00977	2.37345
C16	-0.31084	0.03411	0.38064
C17	-0.29896	0.08394	0.54968
C18	-0.33333	0.07941	0.77697
C19	-1.25516	0.13757	2.46947
C20	-0.3237	0.12801	0.92086
C21	-1.24841	0.18642	2.60056
C22	-1.28155	0.18256	0.83266
C23	0.08255	0.42049	0.00172
C24	0.13385	0.43746	-0.13641
C25	0.16821	0.41469	-0.06261
C26	0.14955	0.3736	0.15203
C27	0.09823	0.35642	0.28819
C28	0.06423	0.37992	0.21451
C29	0.25431	0.36742	-0.16515
C30	0.28578	0.34821	-0.0179
C31	0.32116	0.38069	0.20373
C32	0.32672	0.43372	0.26325
C33	0.29475	0.45253	0.11787

C34	0.25751	0.4188	-0.09228
O35	1.2196	1.43361	0.79061
H36	-1.19361	-0.56128	0.97371
H37	-0.37151	-0.56067	1.14479
H38	-1.2322	-0.39843	1.14036
H39	-0.36593	-0.52606	2.63257
H40	-1.24993	-0.32589	0.94141
H41	-0.3834	-0.4541	2.43165
H42	-1.30104	-0.29693	0.61753
H43	-0.27721	0.03654	0.25083
H44	-0.36751	0.03838	0.84259
H45	-1.22909	0.14163	2.29116
H46	-0.35035	0.12285	1.09859
H47	-1.21904	0.22708	2.51188
H48	0.05678	0.43894	-0.05774
H49	0.14709	0.46866	-0.30142
H50	0.17421	0.35439	0.21718
H51	0.08645	0.32552	0.45271
H52	0.22569	0.34136	-0.32853
H53	0.28169	0.30805	-0.07815
H54	0.35429	0.45948	0.43051
H55	0.29721	0.49223	0.17538

Table S13. Unit cell information and fractional atomic coordinates of TUS-622.

Space group		*P*3	
Calculated unit cell		$a = b = 27.0491$ Å, $c = 4.9291$ Å, $\alpha = \beta = 90^o$, $\gamma = 120^o$	
Measured unit cell		$a = b = 27.0391$ Å, $c = 4.9277$ Å, $\alpha = \beta = 90^o$, $\gamma = 120^o$	
Pawley refinement		$R_p = 3.46\%$, $R_{wp} = 4.58\%$	
Atoms	x	y	z
C1	-1.28375	-0.6118	1.09991
C2	-1.23647	-0.55779	1.03484
C3	-1.24211	-0.5099	0.97867
C4	-1.29617	-0.51453	1.01881
C5	-0.33842	-0.61695	1.11129
C6	-0.34189	-0.56661	1.10634
C7	-1.31042	-0.47085	0.9269
C8	-1.28103	-0.41431	1.02019
C9	-0.353	-0.48626	2.72858
C10	-1.29268	-0.37352	0.91084
C11	-0.36451	-0.44565	2.61873
C12	-1.33383	-0.38867	0.70617
C13	-1.34241	-0.34441	0.57714
N14	-0.37789	-0.35752	2.37619
N15	-0.33773	0.00198	2.38442
C16	-0.28774	0.04767	0.39573
C17	-0.27851	0.09607	0.5677
C18	-0.31314	0.08867	0.79292
C19	-1.23739	0.15129	2.49026
C20	-0.30665	0.13603	0.93723
C21	-1.2337	0.19866	2.62256
C22	-1.26736	0.19199	0.85164
C23	0.09373	0.4352	0.04594
C24	0.13902	0.44489	-0.12409
C25	0.16054	0.40743	-0.12285
C26	0.13506	0.35907	0.04541
C27	0.08972	0.34925	0.21499
C28	0.06896	0.38768	0.21805
C29	0.24591	0.34033	-0.17553
C30	0.27294	0.3201	-0.00351
C31	0.30956	0.35533	0.20418
C32	0.31889	0.4109	0.23169
C33	0.29125	0.43072	0.06144
C34	0.25422	0.39525	-0.1403
S35	1.21774	1.42072	0.64329

H36	-1.19578	-0.55379	0.98862
H37	-0.38137	-0.56742	1.15092
H38	-1.24844	-0.40128	1.1724
H39	-0.37608	-0.52956	2.65173
H40	-1.26871	-0.32985	0.98216
H41	-0.39646	-0.45871	2.46077
H42	-1.31795	-0.301	0.65011
H43	-0.25373	0.05366	0.26086
H44	-0.34567	0.0464	0.85541
H45	-1.21117	0.1575	2.31366
H46	-0.33371	0.12879	1.11194
H47	-1.20653	0.24029	2.53703
H48	0.0773	0.4644	0.04123
H49	0.15725	0.48156	-0.25781
H50	0.14889	0.32793	0.04226
H51	0.07038	0.31084	0.33616
H52	0.21786	0.31299	-0.33411
H53	0.26521	0.27741	-0.03777
H54	0.34736	0.43877	0.3878
H55	0.29837	0.47353	0.08776

12. Supplementary references

(1) P. Wang, X. Chen, Q. Jiang, M. Addicoat, N. Huang, S. Dalapati, T. Heine, F. Huo, D. Jiang, *Angew. Chem., Int. Ed.* **2019**, *58*, 15922–15927.

(2) P. H. Merrell, M. F. Ellis, PREPARATION OF DAMNOEDIPHENYL ETHERS. *U.S. Patent* 4,539,428, Sept 3, 1985.

(3) H. H. Hodgson, *J. Chem. Soc.,Trans.* **1924**, *125*, 1855–1858.

(4) P. Giannozzi, O. Baseggio, P. Bonfà, D. Brunato, R. Car, I. Carnimeo, C. Cavazzoni, S. de Gironcoli, P. Delugas, F. Ferrari Ruffino, A. Ferretti, N. Marzari, I. Timrov, A. Urru, S. Baroni, *J. Chem. Phys.* **2020**, *152*, 154105.

(5) P. Giannozzi, S. Baroni, N. Bonini, M. Calandra, R. Car, C. Cavazzoni, D. Ceresoli, G. L. Chiarotti, M. Cococcioni, I. Dabo, A. D. Corso, S. de Gironcoli, S. Fabris, G. Fratesi, R. Gebauer, U. Gerstmann, C. Gougoussis, A. Kokalj, M. Lazzeri, L. Martin-Samos, N. Marzari, F. Mauri, R. Mazzarello, S. Paolini, A. Pasquarello, L. Paulatto, C. Sbraccia, S. Scandolo, G. Sclauzero, A. P. Seitsonen, A. Smogunov, P. Umari, R. M. Wentzcovitch, *J. Phys. Condens. Matter* **2009**, *21*, 395502.

(6) P. Giannozzi, O. Andreussi, T. Brumme, O. Bunau, M. B. Nardelli, M. Calandra, R. Car, C. Cavazzoni, D. Ceresoli, M. Cococcioni, N. Colonna, I. Carnimeo, A. D. Corso, S. de Gironcoli, P. Delugas, R. A. DiStasio, A. Ferretti, A. Floris, G. Fratesi, G. Fugallo, R. Gebauer, U. Gerstmann, F. Giustino, T. Gorni, J. Jia, M. Kawamura, H.-Y. Ko, A. Kokalj, E. Küçükbenli, M. Lazzeri, M. Marsili, N. Marzari, F. Mauri, N. L. Nguyen, H.-V. Nguyen, A. Otero-de-la-Roza, L. Paulatto, S. Poncé, D. Rocca, R. Sabatini, B. Santra, M. Schlipf, A. P. Seitsonen, A. Smogunov, I. Timrov, T. Thonhauser, P. Umari, N. Vast, X. Wu, S. Baroni, *J. Phys. Condens. Matter* **2017**, *29*, 465901.

(7) J. P. Perdew, K. Burke, M. Ernzerhof, *Phys. Rev. Lett.* **1996**, *77*, 3865–3868.

(8) H. J. Monkhorst, J. D. Pack, *Phys. Rev. B* **1976**, *13*, 5188–5192.

(9) S. Grimme, J. Antony, S. Ehrlich, H. Krieg, *J. Chem. Phys.* **2010**, *132*, 154104.

(10) S. Grimme, S. Ehrlich, L. Goerigk, *J. Comput. Chem.* **2011**, *32*, 1456–1465.

(11) E. C. Kohlrausch, H. A. Centurion, R. W. Lodge, X. Luo, T. Slater, M. J. L. Santos, S. Ling, V. R. Mastelaro, M. J. Cliffe, R. V. Goncalves, J. A. Alves Fernandes, *J. Mater. Chem. A* **2021**, *9*, 26676–26679.

(12) J. W. M. Osterrieth, J. Rampersad, D. Madden, N. Rampal, L. Skoric, B. Connolly, M. D. Allendorf, V. Stavila, J. L. Snider, R. Ameloot, J. Marreiros, C. Ania, D. Azevedo, E. Vilarrasa-Garcia, B. F. Santos, X.-H. Bu, Z. Chang, H. Bunzen, N. R. Champness, S. L. Griffin, B. Chen, R.-B. Lin, B. Coasne, S. Cohen, J. C. Moreton, Y. J. Colón, L. Chen, R. Clowes, F.-X. Coudert, Y. Cui, B. Hou, D. M. D’Alessandro, P. W. Doheny, M. Dincă, C. Sun, C. Doonan, M. T. Huxley, J. D. Evans, P. Falcaro, R. Ricco, O. Farha, K. B. Idrees, T. Islamoglu, P. Feng, H. Yang, R.

S. Forgan, D. Bara, S. Furukawa, E. Sanchez, J. Gascon, S. Telalović, S. K. Ghosh, S. Mukherjee, M. R. Hill, M. M. Sadiq, P. Horcajada, P. Salcedo-Abraira, K. Kaneko, R. Kukobat, J. Kenvin, S. Keskin, S. Kitagawa, K.-i. Otake, R. P. Lively, S. J. A. DeWitt, P. Llewellyn, B. V. Lotsch, S. T. Emmerling, A. M. Pütz, C. Martí-Gastaldo, N. M. Padial, J. García-Martínez, N. Linares, D. Maspoch, J. A. Suárez del Pino, P. Moghadam, R. Oktavian, R. E. Morris, P. S. Wheatley, J. Navarro, C. Petit, D. Danaci, M. J. Rosseinsky, A. P. Katsoulidis, M. Schröder, X. Han, S. Yang, C. Serre, G. Mouchaham, D. S. Sholl, R. Thyagarajan, D. Siderius, R. Q. Snurr, R. B. Goncalves, S. Telfer, S. J. Lee, V. P. Ting, J. L. Rowlandson, T. Uemura, T. Iiyuka, M. A. van der Veen, D. Rega, V. Van Speybroeck, S. M. J. Rogge, A. Lamaire, K. S. Walton, L. W. Bingel, S. Wuttke, J. Andreo, O. Yaghi, B. Zhang, C. T. Yavuz, T. S. Nguyen, F. Zamora, C. Montoro, H. Zhou, A. Kirchon, D. Fairen-Jimenez, *Adv. Mater.* **2022**, *34*, 2201502.

(13) M. Shan, B. Seoane, E. Rozhko, A. Dikhtiarenko, G. Clet, F. Kapteijn, J. Gascon, *Chem. - Eur. J.* **2016,** *22,* 14467–14470.

(14) C. Liang, Y. Zhang, K. Li, X. Li, *J. Mater. Chem. A* **2024,** *12,* 17260–17269.

(15) C. Liang, K. Li, T. Chen, Y. Zhang, J. Yu, Z. Wang, H. Li, X. Li, *Chem. Eng. Sci.* **2025,** *301,* 120801.

(16) Y. Cheng, Y. Ying, L. Zhai, G. Liu, J. Dong, Y. Wang, M. P. Christopher, S. Long, Y. Wang, D. Zhao, *J. Membr. Sci.* **2019,** *573,* 97–106.

(17) Z. Kang, Y. Peng, Y. Qian, D. Yuan, M. A. Addicoat, T. Heine, Z. Hu, L. Tee, Z. Guo, D. Zhao, *Chem. Mater.* **2016,** *28,* 1277–1285.

(18) Y. Pan, X. Zhang, W. He, L. Zheng, X. Han, *Chin. J. Chem. Eng.* **2025,** *77,* 123–134.

(19) Z. Li, G. Fan, M. Chen, Q. Shen, S. Cong, R. Castro-Muñoz, D. Li, J. Wang, Y. Zhang, *J. Membr. Sci.* **2025**, *735*, 124522.

(20) Y. Cui, X. Cui, G. Yang, P. Yu, C. Wang, Z. Kang, H. Guo, D. Xia, *J. Membr. Sci.* **2024**, *689*, 122174.

(21) Y. Liu, C. Wu, Z. Zhou, W. Liu, H. Guo, B. Zhang, *J. Membr. Sci.* **2022**, *659*, 120787.

(22) Q. Zhang, M. Zhou, X. Liu, B. Zhang, *J. Membr. Sci.* **2021**, *636*, 119612.

(23) Y. Zhang, E. Jangodaz, B. H. Yin, S. G. Telfer, *Chem. Commun.* **2024**, *60*, 5924–5927.

(24) C. Dong, Q. Xin, Q. Ma, K. Zhao, Y. Kou, Y. Pan, H. Gao, L. Zhao, X. Ding, Y. Zhang, W. Huang, L. Dong, X. Li, *J. Appl. Polym. Sci.* **2025**, *142*, e57523.

(25) X. Cao, H. Xu, S. Dong, J. Xu, Z. Qiao, S. Zhao, J. Wang, Z. Wang, *J. Membr. Sci.* **2020,** *601,* 117882.

(26) A. R. Nabais, A. P. S. Martins, V. D. Alves, J. G. Crespo, I. M. Marrucho, L. C. Tomé, L. A. Neves, *Sep. Purif. Technol.* **2019,** *222,* 168–176.

(27) N. Liu, J. Cheng, W. Hou, X. Yang, J. Zhou, *J. Appl. Polym. Sci.* **2021,** *138,* 50553.

(28) G. Huang, A. P. Isfahani, A. Muchtar, K. Sakurai, B. B. Shrestha, D. Qin, D. Yamaguchi, E. Sivaniah, B. Ghalei, *J. Membr. Sci.* **2018,** *565,* 370–379.

(29) L. Dong, B. Gao, H. Ma, Y. Liu, Y. Dong, Y. Zhu, Y. Bai, C. Zhang, H. Gao, H. He, H. Meng, *Chem. Eng. Sci.* **2024,** *285,* 119562.

(30) S. Quan, S. W. Li, Y. C. Xiao, L. Shao, *Int. J. Greenhouse Gas Control* **2017,** *56,* 22–29.

(31) J. Cheng, Y. Wang, L. Hu, N. Liu, J. Xu, J. Zhou, *J. Membr. Sci.* **2020,** *597,* 117644.

(32) F. U. Nigiz, N. D. Hilmioglu, *Int. J. Hydrogen Energy* **2020,** *45,* 3549–3557.

(33) A. Jomekian, B. Bazooyar, R. M. Behbahani, T. Mohammadi, A. Kargari, *J. Membr. Sci.* **2017**, *524*, 652–662.

(34) A. S. L. Gouveia, M. Yáñez, V. D. Alves, J. Palomar, C. Moya, D. Gorri, L. C. Tomé, I. M. Marrucho, *Sep. Purif. Technol.* **2021**, *259*, 118113.

(35) M. Klepić, K. Setničková, M. Lanč, M. Žák, P. Izák, M. Dendisová, A. Fuoco, J. C. Jansen, K. Friess, *J. Membr. Sci.* **2020**, *597*, 117623.

(36) S. Ashtiani, M. Khoshnamvand, D. Bouša, J. Šturala, Z. Sofer, A. Shaliutina-Kolešová, D. Gardenö, K. Friess, *Int. J. Hydrogen Energy* **2021**, *46*, 5449–5458.

(37) A. S. L. Gouveia, V. Oliveira, A. M. Ferraria, A. M. B. Do Rego, M. J. Ferreira, L. C. Tomé, A. Almeida, I. M. Marrucho, *J. Membr. Sci.* **2022**, *642*, 119903.

(38) Y. Wu, D. Zhao, S. Chen, J. Ren, K. Hua, H. Li, M. Deng, *Sep. Purif. Technol.* **2021**, *261*, 118243.

(39) S. Dalakoti, A. Jha, N. Singh, A. Arya, R. S. Murali, S. Dasgupta, *J. Polym. Sci.* **2025**, *63*, 4138–4152.

(40) J. Cheng, W. Hou, N. Liu, C. Yang, J. Zhou, *J. Appl. Polym. Sci.* **2022**, *139*, e52840.
